# Automated FDG uptake/PET-CT fused scan diagnosis of various lymph node tumors using object detection AI techniques

**DOI:** 10.1038/s41598-026-53867-9

**Published:** 2026-06-04

**Authors:** Muhammad Abdeltawab, Eman AbdelMaksoud, Amira Samy Talaat, Ahmed Elgarayhi, Mohammed Sallah, Mohammed Elmogy

**Affiliations:** 1 Physics, Faculty of Science, Mansoura University, 35516 Mansoura, Dakahlia Egypt; 2 Information Technology, Faculty of Computers and Information, Arish University, 45511 Al-Arish, North Sinai Egypt; 3 Computers and Systems, Electronics Research Institute, Cairo, 12622 Egypt; 4 Physics, Faculty of Sciences, Bisha University, 61922 Bisha, Saudi Arabia; 5 Information Technology, Faculty of Computers and Information, Mansoura University, 35516 Mansoura, Dakahlia Egypt

**Keywords:** Lymph Nodes (LN), PET-CT Scans, Object Detection, Modified YOLOv8, Mean Average Precision (mAP50), Cancer, Computational biology and bioinformatics, Engineering, Mathematics and computing, Medical research

## Abstract

Lymph nodes (LN) constitute a vital component of the lymphatic system, serving a pivotal role in immune functioning and maintaining fluid balance in the body. Moreover, they serve as markers for tailoring treatments. Inaccurate assessment of LN status may lead to either inadequate treatment or an overly aggressive treatment approach, thereby heightening the risk of recurrence and postoperative complications. Many imaging techniques are used to assess and characterize LNs, but they are limited by low sensitivity for detecting small metastases. Therefore, artificial intelligence (AI) object detection techniques are utilized to localize the relevant objects in images and classify them into relevant classes. This paper proposes a method for detecting 13 LN classes across different body organs using real-world PET-CT datasets. We provide two modules: the first establishes a new LN dataset by fusing CT and PET images for each patient, then denoising and annotating the classes. The dataset was first divided into 80% training, 10% validation, and 10% testing, with data augmentation applied only to the training set to avoid data leakage. Subsequently, 5-fold cross-validation was conducted on the training and validation data to ensure a more reliable evaluation. The final results are reported based on the cross-validation protocol, while the hold-out test set is used for independent assessment. The second module is object detection based on a modified YOLOv8. We selected the kernel, optimized the feature-extraction backbone layers, and tuned other hyperparameters. We compared the performance of eight popular one-stage architectures: YOLOv7, YOLOv8, YOLOv9, YOLOv10, YOLOv11, YOLOv12, YOLONas, and the modified YOLOv8. Work performance has been measured using precision, recall, mean average precision (mAP50), and Dice similarity coefficient (DSC). The findings demonstrate the superiority of the proposed method, with improvements of 78%, 75%, 81%, and 76%, respectively.

## Introduction

The assessment of lymph node (LN) status is paramount in guiding treatment decisions^1^. Evaluating LN status based on radiologists’ judgment for preoperative imaging can be time-consuming and error-prone. Incorrectly assessing LN status can lead to insufficient or overly aggressive treatment, increasing the likelihood of recurrence and postoperative complications^2^. Quantitative radiomic imaging features of tumors have been effectively used in various studies to predict lymph node metastasis^3,4^. These characteristics provide a means to predict the tumor’s lymph node status.

Nonetheless, traditional radiomic techniques require segmentation, feature extraction, and selection, which can be arduous and time-consuming^5^. Moreover, segmentation may be low in reproducibility and susceptible to human error. Thus, there is a pressing need to develop a more accurate, precise, and convenient method for determining LN status. The integration of artificial intelligence (AI) into medical imaging has led to notable progress^6,7^. AI has been employed to identify imaging-based biomarkers that improve disease prognosis, including lung cancer^8,9^, nasopharynx cancer^10^, and gliomas^11^.

Deep learning (DL) technology has made steady progress in recent years, and it has already been the subject of numerous clinical investigations focused on pathological diagnosis and the detection of diseases like lymphoma, brain tumors, prostate cancer, lymph nodes, and lung nodules^12–16^. DL is a fundamental component of AI and has demonstrated remarkable success across various medical imaging tasks. These tasks encompass image segmentation, lesion and landmark identification, registration, fusion, microscopic imaging analysis, computer-aided diagnosis, annotation, and prognosis analysis. DL has proven to be highly effective in classifying and identifying diseases^17–19^.

End-to-end automatic feature extraction operates without human intervention, yielding the most implicit features. Convolutional neural networks (CNNs) heavily rely on these implicit features for their performance, making them vital in numerous medical applications. However, their interpretation can be challenging^20,21^. Conducting unnecessary surgical dissection of lymph nodes when metastatic cervical lymph nodes are not present can increase complications, while delayed dissection for metastases may lead to disease progression. Various imaging techniques, such as ultrasonography (US), computed tomography (CT), magnetic resonance imaging (MRI), and fluorine-18-2-fluoro-2-deoxy-D-glucose positron emission tomography (18F-FDG PET), have been extensively used to evaluate cervical lymph node status in individuals with head and neck (HNC) conditions^22^.

Specific invasive diagnostic methods, such as the combination of fine-needle aspiration cytology (FNAC) and sonography, have been employed to improve diagnostic precision. Nevertheless, the effectiveness of FNAC depends on the expertise of the physicians and the location of potentially suspicious lymph nodes. Furthermore, lymph node swelling can hinder the puncture procedure, resulting in suboptimal tissue sampling of inferior quality. There is increasing demand for readily available, noninvasive diagnostic tools that reliably establish the histological diagnosis of lymphadenopathy or metastases^23^.

Positron emission tomography (PET)/CT, an advanced imaging modality, has been used to diagnose and stage various malignancies, employing fluorine-18 fluorodeoxyglucose (18F-FDG)^4^. In this approach, PET provides functional information about the lymph node’s activity, while CT offers precise anatomical localization^5^. Manual interpretation of PET/CT images has traditionally been considered accurate in distinguishing abnormal nodes based on their distinctive standard uptake values (SUVs)^6^. This manual assessment can effectively determine nodal status for lymphoma involvement or metastases from solid malignancies, particularly in cases where highly malignant solid tumors exhibit strong metabolic activity in lymph nodes.

Nevertheless, there are inherent difficulties, as diagnostic precision is contingent on radiologists’ experience when evaluating moderately active lymph nodes. Consequently, developing an automated prediction model to identify lymph nodes in PET/CT images is imperative. This model could assist in the histological diagnosis of enlarged cervical lymph nodes, enabling the identification of pathological components within the targeted nodes. This applies regardless of whether they are associated with lymphomas or metastases from solid tumors and relies on multidimensional imaging patterns^23^.

An inherent limitation of auto-segmentation is its reliance on the algorithm’s accuracy in aligning the anatomies of diverse patients, using a single atlas for each new patient. This dependency may lead to inaccuracies due to variations in patient anatomy and limited contrast in lymph node regions on CT scans. To mitigate these inaccuracies, the authors proposed adopting a multi-atlas-based auto-segmentation approach, which can capture a broader spectrum of anatomic variations and enhance the quality of the resulting segmentations^24^.

Numerous studies, including our own, have demonstrated that quantitative radiomic features extracted from tumor images can serve as predictive biomarkers for LN status^7–9^ and have been effectively utilized to predict LN metastasis^10^. However, traditional radiometric techniques can require significant labor and time, including tasks such as segmentation, feature extraction, and selection^10^. Additionally, segmentation might exhibit limited repeatability and be vulnerable to human error. Hence, there is an urgent need to develop a more objective, precise, and user-friendly approach to accurately assess lymph node status^13^.

Over the past few years, DL has achieved remarkable progress in identifying and categorizing diseases^12,13,25^. Primarily leaning on image scans, these studies frequently overlook essential clinical features like age, gender, molecular markers, genomics, and smoking history. Only a limited number of investigations have integrated previous clinical knowledge into DL frameworks, as demonstrated by Xie et al.^14^. They proposed a collaborative DL method based on knowledge to classify benign and malignant lung nodules. Nevertheless, this knowledge mainly originates from CT images, including shape and texture features, with limited incorporation of additional clinical information. Additionally, when previous clinical features were integrated solely through logistic regression to predict the risk of lymph node metastasis, performance was suboptimal, yielding an area under the curve (AUC) of 79.6%^26^.

**Fig. 1:**
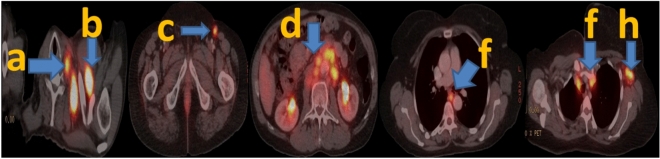
Different lymph node tumor cases with different levels: (**a**) Cervical LN, (**b**) Supraclavicular LN, (**c**) LT Inguinal LN, (**d**) Abdominal LN, (**f**) Mediastinal LN, and (**h**) LT Axillary LN.

To achieve precise staging, researchers have applied statistical analysis and machine learning (ML) techniques to uncover intricate relationships between comprehensive patient characteristics and the status of lymph node metastasis (LNM)^8,10,27^. As hospital information systems have advanced rapidly, a substantial volume of electronic medical records (EMRs) has become accessible, encompassing nearly all clinical information about patients. However, specific critical data points are embedded within unstructured narrative sections, presented in free-text format. These details include tumor size, lymph node characteristics, tumor density, and pleural involvement, making their direct utilization challenging. Manually extracting this information is time-consuming and error-prone^28^. Figure 1 shows some examples of LN.

The key contributions of this study can be outlined as follows:

- The development of a cutting-edge DL framework designed for the simultaneous identification of interwoven LN in diverse PET-CT images. To evaluate the proposed framework, we tested it on a recent, exclusive dataset encompassing a broad spectrum of intricate LN across 15 organ categories in the human body.
- Establishing a dataset by collecting CT and PET images, preprocessing, fusion, annotation, and augmentation.
- Evaluating the effectiveness of our proposed framework by comparing it with some state-of-the-art models and existing approaches, utilizing five different metrics. The experimental outcomes clearly illustrate the practicality and superior performance of our framework.
- We adapted and modified the YOLOv8 approach that detected 13 LN classes from 15 LN annotated classes across various organs throughout the body.

The remainder of this manuscript is organized into five sections. Section Related work explores existing research, identifies current limitations, and highlights the primary approaches and solutions integrated into the proposed system to address these challenges. Section Methods outlines the specific stages and methods employed within the proposed framework. In Section The results, a comprehensive overview of various experiments and their corresponding findings is presented. The discussion commences in Section Discussion, providing a comparative analytical examination of the proposed system alongside other state-of-the-art methodologies. Finally, Section Conclusion summarizes our work and findings and suggests directions for future research.

## Related work

In this section, we provide an overview of recent studies on LN detection. The content of Table 1 offers a concise literature review summary. For example, Courot et al.^29^ detected and showed CT segmental lymphadenopathy of the head and neck. Training was performed on 117 CT images, and testing was performed on 150 CT images. The suggested model achieved a mean score of 63%. The small amount of available training data limited the model. Li et al.^30^ developed a deep learning nomogram (DLN) incorporating a deep learning signature and clinical risk factors to predict lymph node metastasis in 418 patients with cervical cancer, achieving an AUC of 0.925. The outcomes were biased due to the limited sample size, and the correlation between the deep learning nomogram (DLN) and specific essential proteins associated with LNM in cervical cancer was overlooked.

Tomita et al.^31^ evaluated deep learning for the preoperative diagnosis by comparing normal and malignant cervical LNs with the Xception DL method, achieving an AUC of 89.8% at levels I–II and 96.7% at level II. The model needed further investigation to determine the optimal technique for diagnosing cervical LN status. Yang et al.^32^ classified lymphoma and cervical LNM with ResNet50, which had an accuracy of 78.13%, and the DL-SVM model had an accuracy of 86.96%. The study needs to include more histology elements, such as metastases, lymphomas, infections, and tuberculosis.

Zhang et al.^26^ classified enlarged cervical lymph nodes in 276 patients using a CNN filtered with LASSO, achieving an accuracy of 87.50%. They excluded individuals with specific pathological subgroups and had limited sample sizes for several disorders. Ariji et al.^33^ identified cervical lymph nodes in 56 patients with oral squamous cell carcinoma utilizing the DetectNet model. The recall for detecting metastatic and non-metastatic nodes was 90% and 80%, respectively. The system overlooked small lymph nodes with metastasis due to their limited number. Pham and Tuan^34^ predicted LN malignancy in 148 patients with primary lung cancer using AlexNet, DenseNet-201, and SqueezeNet. The classification accuracies for SqueezeNet, AlexNet, and DenseNet201 were 93%, 96%, and 100%, respectively. Few patients with lung cancer participated in the trial.

Iuga et al.^35^ used a 3D full CNN to detect and segment thoracic LNs in 90 patients, achieving a detection rate of 76.9%. A single radiologist segmented the datasets, which were presumed homogeneous. Meng et al.^36^ detected mediastinal and abdominal LN for 90 mediastinal patients and 86 abdominal patients with sensitivities of 94% and 96% for the mediastinum and abdomen, respectively. It was difficult to identify all the lymph nodes. Dong et al.^25^ predicted the quantity of LN metastases in gastric cancer that is locally advanced for 730 patients using a DL radiomic nomogram (DLRN). The metastasis rate from LN in the validation cohorts was 0.822. For generalizability, studies of large populations of non-Asians should be examined. Accuracy might be increased by combining CT and endoscopic ultrasonography.

Li et al.^37^ forecasted stage II colorectal cancer (CRC) in a group of 230 patients employing the CNN technique. The disease-free and overall survival predictions improved to AUCs of 76% and 91%, respectively. The limited dataset led to overfitting. Additionally, no tumor markers, clinical information, or genetic characteristics were available in the training set. Fan et al.^38^ projected the lymphovascular invasion (LVI) status of gastric cancer in a cohort of 101 patients using AdaBoost, linear discriminant analysis (LDA), and LR, achieving AUC values of 94.4%, 92.9%, and 92.1%, respectively. The study had multiple limitations. First, it included only patients from a single research institute, and the sample size was limited. Second, there was a need to explore the significance of radiomic features extracted from multiphasic contrast-enhanced CT scans, alongside other clinical factors. Finally, there was a requirement to develop a deep learning technique for automated gastric cancer segmentation.

Jin et al.^39^ utilized the ResNet-18 model to predict LNM in gastric cancer, yielding a sensitivity of 74.3% and a specificity of 93.6%. The study had limited information on lymph nodes due to their proximity to the hilum of the spleen. The models were developed and evaluated using data from a single ethnic population. Liu et al.^40^ achieved a best-performing accuracy of 90.88% for 401 breast cancer patients using a deformable attention VGG19 (DA-VGG19) model for predicting axillary lymph node metastasis. Additional multi-center studies are necessary to enhance the model’s robustness and reproducibility. The study presented several limitations: firstly, it relied on a 2D rather than a 3D examination. Secondly, lesions with diameters less than 0.5 cm were excluded. Lastly, further multi-center studies are needed to assess the model’s robustness and reproducibility.

Song et al.^41^ predicted axillary LNM for 100 patients with the XGBoost algorithm. The model accuracy was 80%. The model was biased due to the small number of patients, and all patients who had received any other treatment prior to surgery were excluded. Sabbagh et al.^42^ developed an XGBoost model that outperformed traditional nomograms with an AUC of 0.82, showing a significant improvement (95% CI for DAUC: 0.042–0.12) over the Roach formula.

Jin et al.^43^ developed a predictive model for bone metastasis using a score derived from the grey-level co-occurrence matrix (GLCM) to aid clinical diagnosis and treatment. 614 CRC patients got pelvic multiparameter MRIs. The GLCM-based score and ML algorithms, including the ANN, RF, DT, and SVM, were utilized to develop a predictive model for bone metastases in patients with CRC. The RF model achieved an AUC of 92.6%, with a 95% confidence interval of 0.873-0.979. However, there was no discussion of bone anatomy within a single pelvic cavity or of the specific focal level.

Bütün et al.^44^ used the PCAM dataset, comprising 220,025 labeled lymph node images, to achieve a classification accuracy of 98.60% with ResNet-101 and a 1-cycle learning-rate policy. Wang and Zhang^45^ proposed an approach to predict tumor LNM by integrating Wasserstein distance-based generative adversarial networks with a neural network optimized via particle swarm optimization (WGAN-psoNN). They applied the method to many datasets. The result achieved 94% accuracy on the BLCA dataset. A few lncRNA samples were connected to each cancer.

As discussed in previous studies, the limitations of the current related work can be summarized as follows. First, limited patient numbers and sample sizes, as well as the number of lymph nodes evaluated, may introduce bias. This limits the model’s effectiveness. Limited datasets may lead to overfitting during training. Second, the RoIs were manually described. Drawing RoIs manually requires a pathologist’s guidance. They may not capture all LN traits. Third, some studies used training and validation data sets consisting of LNs with short-axis diameters, cautiously applying the DL algorithm to smaller lymph nodes until validation. No consideration was given to LNs with diameters greater than 8–10 mm. Fourth, some studies share datasets. Factors such as the CT imaging protocol and patient characteristics may introduce bias into the data, since they are derived from the same institution. Consequently, an external validation study is essential to verify the diagnostic performance and applicability of this single-center investigation. Fifth, some studies did not include clinical features in their ML models to enhance performance. Finally, some studies used 2D analysis instead of 3D. Other studies used data from one ethnic group.

To overcome these limitations, we collected 1930 CT and PET images from the same patients and fused them into PET-CT color images. We applied some processing steps to the fused image to remove noise and artifacts. To avoid overfitting, we applied simple data augmentation, as illustrated in the next section. On the other hand, we predicted about 13 classes of various LNs from different organs in the body. The physician manually marked 15 classes in all images and evaluated the annotation of various LN classes. We utilized various matrices for assessment and evaluation.

**Table 1:** The summary of some current related work.

Author	location	Methodology	Patients	Aim	Accuracy	Limitations
Courot et al.^29^	Cervical	U-Net	267	Detect LN from head and neck	Mean score: 63%	Limited training data, Sparse annotations
Cardenas et al.^24^	HNC	U-Net	71	LN CTV contours	median Dice Similarity: 90%, 90%, 89%, and 81% for Ia-V, Ib-V, II-IV, and RP respectively	Pattern variations
Li et al.^30^	Cervical	CNN, and LR	418	Predicting cervical	AUC = 0.925	Limited patient sample
Tomita et al.^46^	Cervical	Xception	39	classify benign and metastatic	AUC 89%,96% for I–II, II	Investigation required
Yang et al.^32^	Cervical	DL-CNN, ResNet50	165	Classify metastasis and lymphoma	ResNet50 AUC = 0.845, accuracy = 78.13% DL-SVM, ResNet50 AUC = 0.901, accuracy= 86.96%, sensitivity= 76.09%, specificity= 94.20%.	Single center dataset, fewer histological components, future assessment, and technique improvements are necessary
Zhang et al.^26^	Cervical	CNN, LASSO	276	Classifying cervical	Accuracy= 87.50%	No abstract features of NN, small sample sizes
Ariji et al.^33^	Cervical	DetectNet	56	Detecting cervical	Recall of metastatic= 90%, non-metastatic= 80%	Small LN numbers
Pham and Tuan^34^	Mediastinal	AlexNet, SqueezeNet, and DenseNet-201	148	classify mediastinal LN malignancy	SqueezeNet=93%, AlexNet=96%, DenseNet201=100%	small Samples, Fuzzy Recurrence Plot (FRPs) are symmetric
Iuga et al.^35^	Thoracic	A 3D fully CNN (u-Net)	90	3D LN detection and segmentation	Total detection rate of 76.9%	segmentation by only one radiologist
Meng et al.^36^	Mediastinal and abdominal LNs	Adaptive Multi-scale Network (AMsNet)	176	LN detection and analysis	Sensitivity 94%, 96% for Mediastinum, abdomen	LNs are not labeled

Author	location	Methodology	Patients	Aim	Accuracy	Limitations
Dong et al.^25^	Gastric cancer	Deep Learning Radiomic Nomogram (DLRN)	730	Predict LNM number	validation cohorts was 82.2%	Asian populations only, not combine ultrasonography, CT
Li et al.^37^	Colorectal cancer	CNN	230	prediction for stage II colorectal	AUCs: 0.76± 0.08 and 0.91± 0.05	Limited data set, not all clinical information, markers, and genetic characteristics were combined
Fan et al.^38^	Gastric cancer	AdaBoost, LDA, LR	101	Predicting LVI status	AUC 94%, 92%, 92% for AdaBoost, LDA, LR	limited sample size, extracted features requires investigation
Jin et al.^39^	Gastric cancer	ResNet-18	1699	Prediction of LNM	AUC = 0.876, sensitivity 74.3%, and specificity 93.6%.	Potential selection biases, data of one ethnic population
Liu et al.^40^	ALN- breast cancer.	DA-VGG19	401	Predict the metastasis	accuracy 90.8%	study based on 2D analysis, short diameter lesions were excluded
Song et al.^41^	ALN metastasis	XGBoost	100	axillary Prediction	Sensitivity 90.9%, specificity 71.4%, accuracy 80%	Small number of patients, fixed threshold
Sabbagh et al.^42^	Pelvic, lymph node involvement (LNI)	two LG models and XGBoost	2563	predicting LNI	AUC 82%, 95%	not include MRI or prostate-specific membrane antigen positron emission tomography data
Jin et al.^43^	pelvic bone metastasis	Gray level Co-occurrence Matrix (GLCM), ANNM, RFM, DTM, and SVMM	614	build a bone metastasis prediction	RFM AUC 92%, 95% CI= 0.873-0.979	the bone structure of a single pelvic cavity is not clear
Bütün et al.^44^	metastasis	ResNet-34/50/101	220,025	detect metastasis	accuracy 98.6%	No other source domains available
Wang et al.^45^	LNM	WGAN-psoNN, KNN, WGAN-KNN	160	LNM Prediction	WGAN-psoNN accuracy = 94%	limited number of lncRNA samples

## Methods

In this segment, we introduce the proposed framework utilizing YOLOv8. The framework comprises two main modules: dataset establishment and modified YOLOv8 modules. Further elaboration on these modules will be provided in the subsequent subsections.

### Dataset establishing module

In this retrospective study, a total of 1,931 PET-CT images were collected from 190 patients diagnosed with lymphoma or lymph node tumors. The patient cohort ranged in age from [40–65] years, comprising 60% male patients and 40% female patients. The imaging data were collected retrospectively from five affiliated imaging centers. Although the data were acquired from multiple centers, all scans followed a standardized imaging protocol using the same PET-CT system (GE PET-CT Discovery IQ). This is to ensure consistency in acquisition parameters across all sites using a GE PET-CT Discovery IQ system following a standardized imaging protocol. The reconstruction slice thickness was set to 1.25 mm, and a standard reconstruction algorithm (Mac) was applied to generate high-quality fused PET-CT images. To improve image quality, Radiant DICOM Viewer was used for preprocessing to reduce noise and artifacts prior to annotation. Although most images were acquired in the axial plane, additional coronal views were included to enhance anatomical coverage and diagnosis.

This study was conducted in accordance with the ethical principles outlined in the Declaration of Helsinki, in addition to receiving formal approval from the Ethics Committee of the Faculty of Science, Mansoura University (Approval Code: Sci-Phy-M-2022-82; issued January 30, 2021). Given the retrospective nature of the study, the Institutional Review Board (IRB) of the Mansoura Faculty of Medicine waived the requirement for informed consent. The data was collected anonymously from March 1, 2022, to April 30, 2023.

**Fig. 2:**
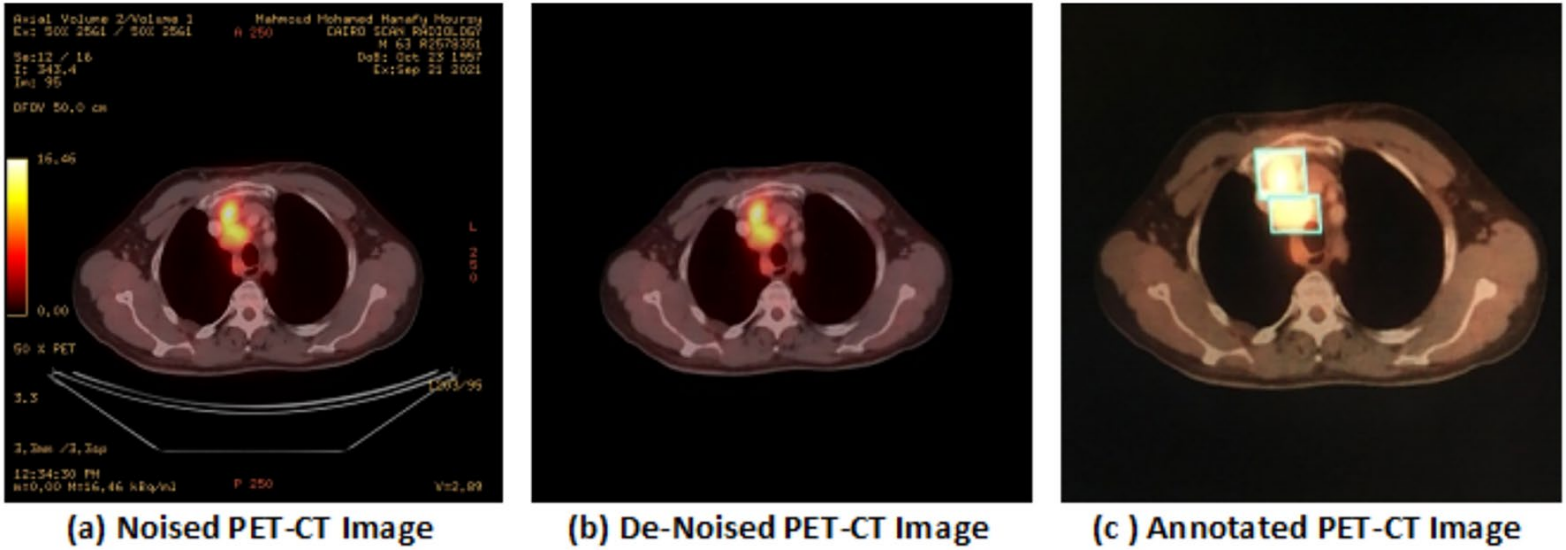
The noise removal step of PET-CT images.

The imaging data were initially stored in the Digital Imaging and Communications in Medicine (DICOM) format and subsequently converted into JPEG format for downstream processing. To enhance data quality and mitigate potential sources of bias, noise, and irrelevant artifacts, the data was rigorously filtered. A dedicated noise-reduction pipeline was applied to all PET-CT images, followed by spatial normalization by resizing to 640$\times$640 pixels. Subsequently, a total of 15 classes were meticulously annotated across the entire dataset. Figure 2 illustrates the preprocessing workflow, including noise removal and annotation procedures.

To address class imbalance and ensure statistical robustness, each class was standardized to 168 images, except for the normal class, which comprised of approximately 192 images.

#### Dataset annotation

All patient data included in this study were obtained from official medical records and imaging reports, ensuring the reliability of the clinical diagnosis. The dataset annotations were supported by clinical PET-CT reports and validated by experienced consultant physicians. The original diagnoses were based on standard clinical reporting performed by board-certified radiologists and nuclear medicine specialists. The images were then reviewed and annotated in alignment with these clinically validated reports to ensure reliable ground truth labeling.

The annotation was performed collaboratively by two experienced specialists with approximately five years of experience in nuclear medicine and PET-CT imaging. Both annotators worked jointly and resolved disagreements through consensus discussion. Due to this collaborative annotation design, in which both annotators labeled the images simultaneously, inter-observer agreement could not be independently measured using Cohen’s kappa coefficient. This methodological choice ensured consistent labeling but precluded statistical assessment of agreement.

Together, physicians with expertise in radiology and oncology independently reviewed all PET-CT scans. Each physician performed a case-by-case examination to confirm the presence, location, and classification of lymph node tumors, and the reports included two opinions: the radiologist’s and the nuclear medicine MD expert. Furthermore, during the annotation stage, a second radiologist, in addition to the previously mentioned radiologist, reviewed and revised all annotated images.

To enhance annotation accuracy:

1. Consensus and Conflict Resolution: In cases of disagreement among physicians, a consensus meeting was held to resolve discrepancies and finalize labels.
2. Ground Truth Verification: Annotated labels were cross-checked against the available histopathology reports whenever accessible, providing an additional layer of validation.
3. Annotation Reliability: Inter-observer agreement was evaluated using Cohen’s k statistic, ensuring consistency across multiple annotators. This rigorous annotation protocol ensures that the dataset contains accurate, reliable labels, providing a solid foundation for training and evaluating the proposed deep learning framework.

#### Data augmentation and validation

The dataset was initially divided into 80% training, 10% validation, and 10% testing sets to obtain baseline performance results. Data augmentation was applied exclusively to the training set, while the validation and test sets were not augmented to prevent data leakage. To ensure a more robust evaluation, a 5-fold cross-validation was subsequently performed on the entire dataset. In each fold, data augmentation was applied only to the training portion.

The primary evaluation results reported in this study are based on the 5-fold cross-validation protocol, while the hold-out split results are provided for comparison, and we recorded that comparison. Crucially, data augmentation was applied strictly to the training subset in each fold after the split. All augmentation techniques were implemented on the fly only for the training samples. The validation and test sets remained in their original, non-augmented state. This protocol ensures that no derivative of a validation or test sample was ever seen during the training phase, thereby eliminating the risk of data leakage.

**Fig. 3:**
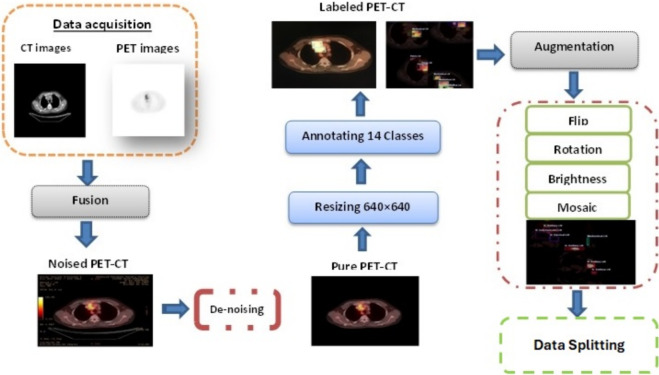
The steps of the dataset establishing phase.

We used simple augmentation methods to increase the number of images in the training set only, preserve annotations, and train the model to learn broader features. Augmentation helps address class imbalance and avoid overfitting. We applied horizontal and vertical flipping, 45$^{\circ }$ clockwise and counterclockwise rotation, and brightness between -40% and +40%. The number of images after augmentation is 4608. Table 2 shows the augmentation parameters. The steps of the dataset-establishing phase are shown in Fig.  3.

**Table 2:** Examples of (On the Fly) data augmentation parameters on Training Dataset.

Data augmentation technique		Settings
Color variation	Brightness	0.4
Scale	Zoom Range	0.5
Geometric transformation	Rotational	$45^{\circ }$
	Flipping	**left-right**

### The modified you only look once version 8 (YOLOv8) module

The YOLO algorithm utilizes a single neural network to simultaneously predict bounding boxes and class probabilities for all objects in an image, making it an object detection method. In this context, YOLOv8 represents the most recent iteration of the YOLO algorithm. YOLOv8 consists of a CNN with two primary components: the backbone and the head^47^. Moreover, the neck serves as a crucial connection between the spine and the head.

The spine consists of 53 convolutional (CONV) layers and employs cross-stage partial connections to improve information flow among these layers. It can be described as an altered rendition of the CSPDarknet53 structure. The backbone architecture relies on a feature pyramid network (FPN), which serves as a feature extractor, accepts an image of any size at a single scale, and produces feature maps at multiple scales, each scaled proportionally, using a fully convolutional approach. It is a universal solution for constructing feature pyramids within deep convolutional networks (DCNs) for various applications, including object detection^48^.

**Fig. 4:**
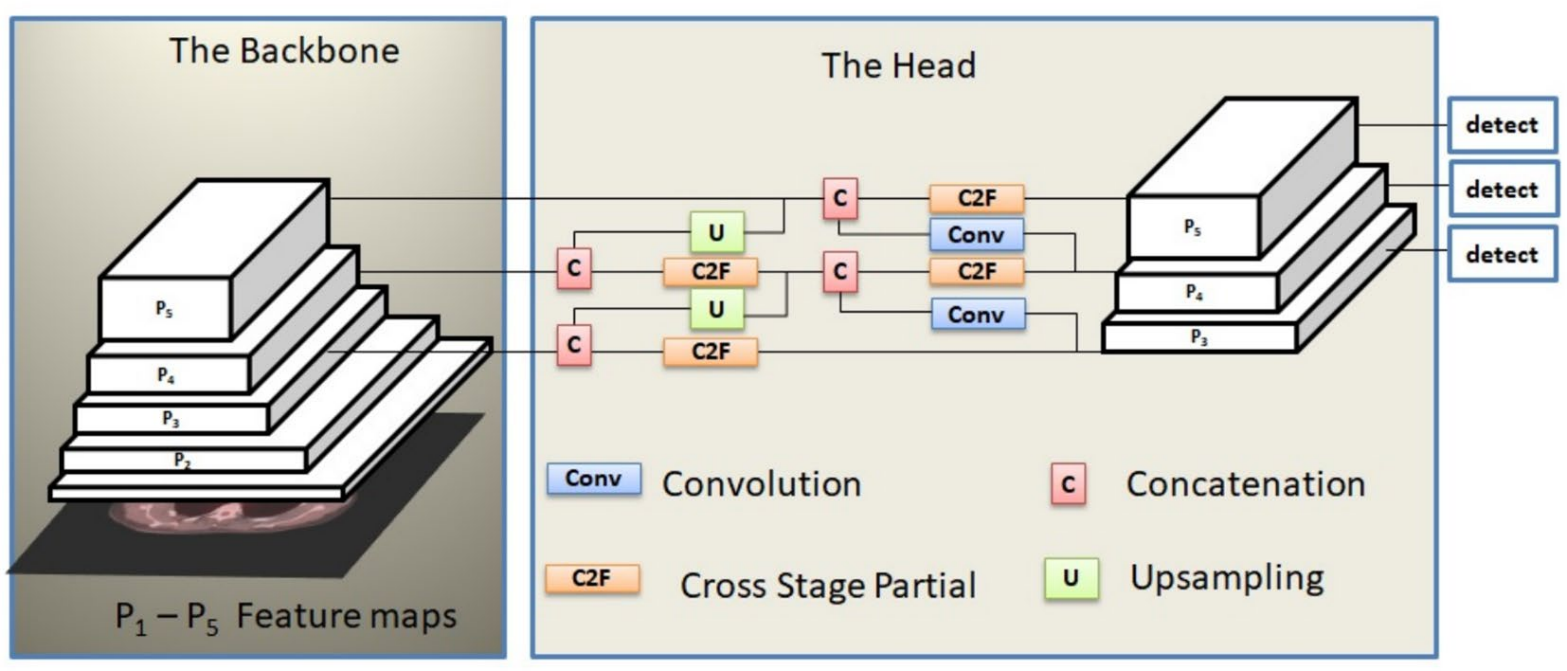
The YOLOv8 architecture.

The YOLOv8 neck builds feature pyramids by amalgamating feature maps extracted from the backbone stages. It integrates features at multiple scales to ensure the network can effectively detect objects of varying sizes^49^. The YOLOv8 head consists of several convolutional layers and a series of fully connected layers. These layers predict bounding boxes, objectness scores, and class probabilities for the objects recognized in an image.^50^. YOLOv8 architecture is shown in Fig. 4.

#### YOLOv8 backbone modification

As YOLOv8 is an advanced open-source model, there is continued interest in maximizing accuracy through diverse techniques. Consequently, we have introduced modifications to the YOLOv8 backbone, detailed below:

- Kernel size becomes $7\times 7$ rather than $3 \times 3$. As a filter, the kernel plays a crucial role in shaping the feature map and determining the convolutional kernel size. In a CNN, the filter size denotes the dimensions of the receptive fields, or ’windows,’ used by the network to analyze input data. Enlarging the filter size in a CNN can enhance accuracy in several ways. First, it reduces parameter sharing, providing the model with greater flexibility. A larger filter size allows for more parameters that are not shared across layers, meaning the weights corresponding to each neuron can be adjusted independently of other neurons in the same layer. This increased flexibility enables better capturing of complex patterns from input data, leading to improved performance. Second, larger filters allow for deeper networks, as they can capture more information from an image in a single pass than smaller filters. This aspect enhances accuracy by providing a more comprehensive representation of the image’s features. Moreover, using large filters instead of stacking many layers with small filters has been shown to work better–large filters have fewer parameters and thus require fewer computations than deep architectures with multiple levels of small feature detectors. Therefore, we tested kernel sizes of 3, 5, 7, and 11. A kernel size of $7\times 7$ yields good performance.
- Deeper networks acquire greater feature information, improving dense prediction. Nonetheless, excessively deep networks can degrade the precision of object location information, and excessive convolutional operations may lead to information loss, particularly for small objects. In this respect, we added 6 Conv blocks rather than 5. Additionally, we increased the number of filters per layer.
- Hyperparameter optimization: some hyperparameters influence the model performance, such as the optimizer that determines how the model updates its weights and the learning rate, which controls how much to adjust the model in response to the estimated error each time the model weights are updated (initial learning rate (lr0) and final learning rate (lrf)). We are referring to the learning rate at the start and end of training. These are applied to the whole network. The batch size dictates the quantity of samples processed before the model adjusts its weights. The hyperparameters are shown in Table 4.

**Fig. 5:**
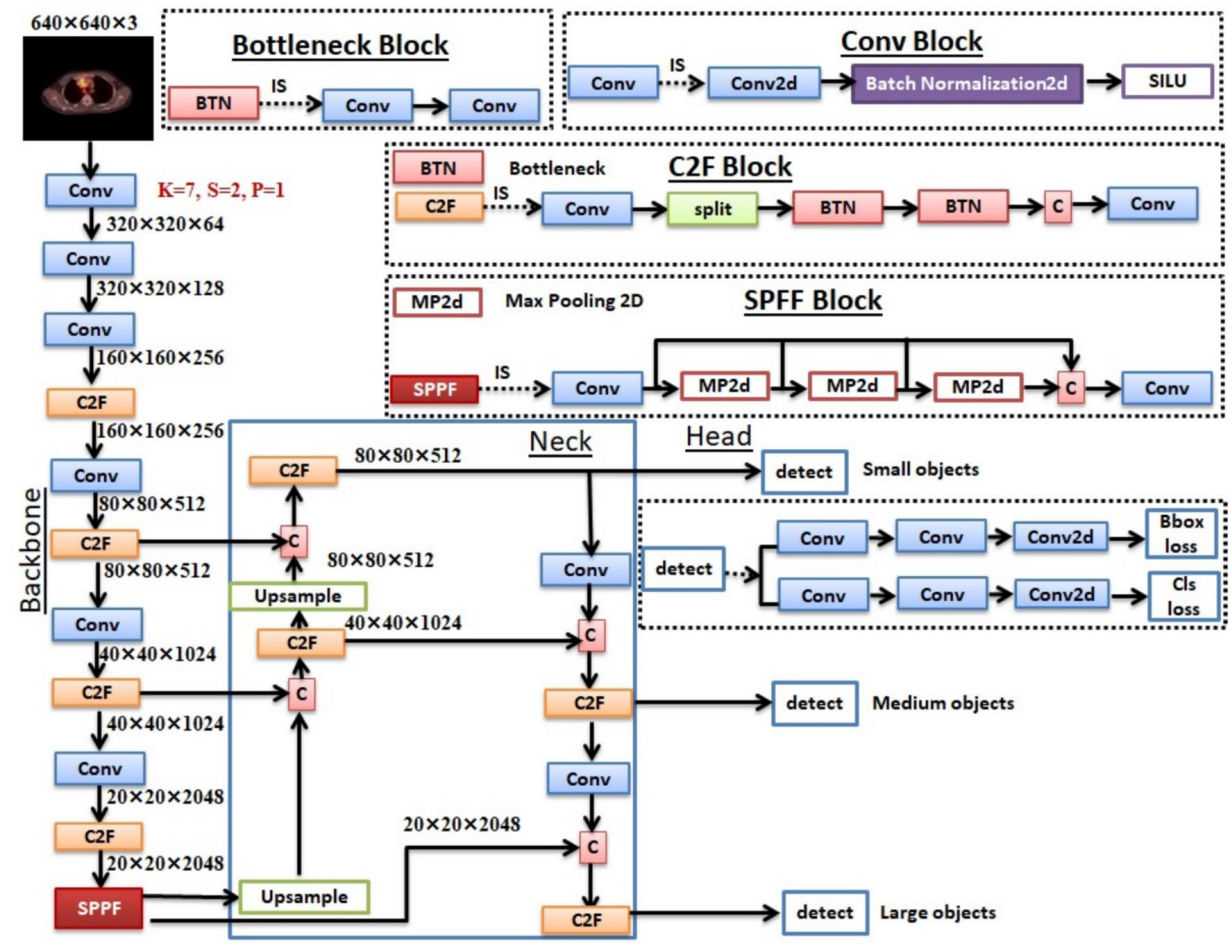
The modified YOLOv8 backbone architecture used in the proposed framework.

Figure 4 shows the YOLOv8 architecture, and Fig. 5 shows the modified YOLOv8 backbone. The backbone consists of three CONV blocks with kernel (K)=7, stride (S)=2, and padding (P)=1. Then, one coarse-to-fine (C2F) block is followed by three Conv blocks, then another C2F block. Finally, spatial pyramid pooling fusion (SPPF) is a refined iteration of spatial pyramid pooling (SPP) that requires fewer floating-point operations (FLOPs). Each CONV block consists of a Conv2D layer followed by a 2D batch normalization layer. The C2F block consists of one Conv block, then a split and bottleneck block, concatenation, and finally, one Conv block. The bottleneck block includes two Conv blocks. The SPPF block includes one Conv block, three concatenated max-pooling layers, and another Conv block. SPPF seeks to comprehensively represent input feature maps across multiple scales. By pooling at different scales, the model can capture features at multiple levels of abstraction, which is particularly advantageous in object detection tasks where recognizing objects of different sizes is crucial.

Table 3 shows the modified YOLOv8 backbone architecture summary. Given an input resolution of 640$\times$640, the proposed backbone generates hierarchical feature maps at strides 2, 4, 8, 16, 32, and 64. The corresponding feature map resolutions are 320$\times$320, 160$\times$160, 80$\times$80, 40$\times$40, 20$\times$20, and 10$\times$10, respectively. The backbone progressively increases the channel depth from 64 to 2048 while employing C2f modules for feature refinement and an SPPF layer at the deepest stage to enhance the receptive field.

**Table 3:** The modified YOLOv8 backbone architecture summary.

Layer	From	Operation	Kernel Stride	**Output Size (H**$\times$**W**$\times$**C)**	Stride	Notes
0	Input	Image	–	640$\times$640$\times$3	1	Input
1	-1	Conv	7$\times$7 2	320$\times$320$\times$64	2	P1
2	-1	Conv	7$\times$7 2	160$\times$160$\times$128	4	P2
3	-1	Conv	7$\times$7 2	80$\times$80$\times$256	8	P3
4	-1	C2f $\times$3	–	80$\times$80$\times$256	8	Feature refinement
5	-1	Conv	7$\times$7 2	40$\times$40$\times$512	16	P4
6	-1	C2f $\times$6	–	40$\times$40$\times$512	16	Deep features
7	-1	Conv	7$\times$7 2	20$\times$20$\times$1024	32	P5
8	-1	C2f $\times$6	–	20$\times$20$\times$1024	32	High-level semantics
9	-1	Conv	7$\times$7 2	10$\times$10$\times$2048	64	P6
10	-1	C2f $\times$3	–	10$\times$10$\times$2048	64	Very deep features
11	-1	SPPF	k=5	10$\times$10$\times$2048	64	Multi-scale pooling

The modified model employs larger convolutional kernels (7$\times$7), an extended detection scale (P6) for large-object detection, and increased channel depth up to 2048 to enhance the representation of deeper semantic features. These design choices improve the model’s ability to detect large and complex objects in the dataset. However, this improvement comes at the cost of increased computational complexity, resulting in higher FLOPs and memory consumption. The primary objective of the proposed model is to achieve more accurate and reliable object detection on the target dataset. In future work, we plan to explore optimizations and modifications of state-of-the-art architectures to address the computational limitations introduced by the current design while maintaining or improving detection performance.

#### YOLOv8 neck modification

Due to modifications to the backbone, the neck is also modified accordingly. In YOLOv8, the SPPF block output is upsampled and concatenated with a third C2F block of the backbone, followed by another C2F block. Then, the C2F block output is upsampled again, concatenated with the backbone’s second C2F block, followed by another C2F block and the Detect1 block in the head architecture. This Detect1 block consists of two Conv blocks followed by one Conv2D layer. Identifying small objects effectively results in losses in bounding box (Bbox) and classification (Cls). The bounding box loss function computes the variance between the predicted bounding box and the actual bounding box^51^.

In return for the neck structure, the last C2F block is followed by a Conv block, which is then concatenated with its previously upsampled C2F block. Then followed by the C2F block, then the detect2 block in the head architecture to detect medium-sized objects, with resulting Bbox loss and Cls loss. After the second detection, the C2F block is followed by the Conv block, concatenated with the SPPF block, and then by the C2F block, which the Detect3 Block follows in the Head architecture to detect large objects. Algorithm 1 shows the steps in detail.

**Algorithm 1:**
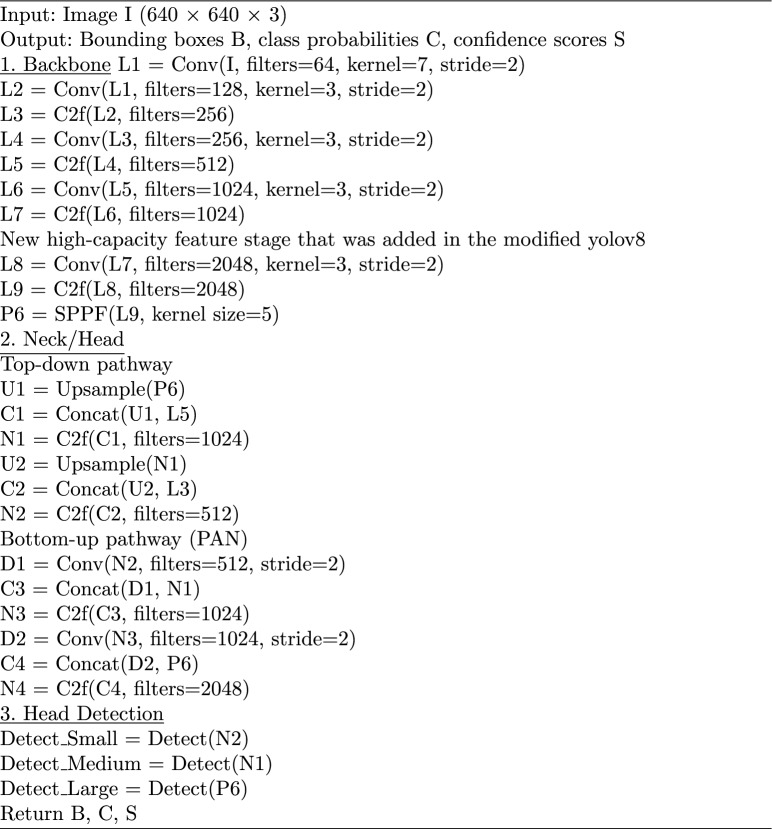
The Modified Yolov8.

**Table 4:** The modified YOLOv8 hyperparameters.

**Parameter**	**Value**
optimizer	Adamax
lr0	$5 \times 10^{-3}$
lrf	$5 \times 10^{-3}$
momentum	0.937
weight decay	0.0005
batch size	16
epochs	200
image size	$640\times 640$

## Lymph nodes establishing 2023 (LYE23) dataset description

The LYE23 dataset contains 1931 PET/CT DICOM images across 15 LN categories. These categories consist of abdominal LN, cervical LN, left-subclavicular LN, Lt-inguinal LN, mediastinal LN, normal, nose LN, pelvis LN, retroperitoneal LN, Right-cervical LN, Right-subclavicular LN, Right-axillary LN, Right-inguinal LN, Left-axillary LN, and Lt-cervical LN. Each class is about 168 images, except for the nose LN and the retroperitoneal LN. However, the normal class is 170 images. The dataset is split into 80% training, 10% validation, and 10% test sets.

The labels folder includes text (txt) files, one for each image. Each .txt file contains the bounding-box coordinates for the object of interest. Each image may include one, two, or three classes. Therefore, the text file may include more lines of directions. In radiographic images, LNs are small, ellipsoid structures measuring 0.1-2.5 cm in maximum length. LN imaging is a useful technique for determining whether nodes are benign or malignant. Axillary lymph nodes, located in the armpit region, comprise five groups: lateral (humoral), anterior (pectoral), posterior (subscapular), central, and apical nodes. Typically numbering between 20 and 49, these nodes drain lymph from the lateral quadrants of the breast, superficial lymph vessels of the chest and abdomen above the navel, and vessels of the upper limb. Categorized by their position in the armpit, they play a crucial role in breast cancer diagnosis and staging, as metastases from breast cancer to axillary lymph nodes are significant indicators of disease progression.

Supraclavicular lymph nodes, located just above the collarbones on both sides of the body, may enlarge due to an immune response to infection or metastatic cancer. Typically numbering between 4 and 12 nodes, they reside superior to the clavicle, alongside the transverse cervical artery, and anterior to the anterior and middle scalene muscles. As a crucial component of the terminal lymphatic system, these nodes receive lymphatic drainage from various regions, including the head, neck, thorax, and abdomen, through an intricate pathway. Treatment for swollen supraclavicular lymph nodes involves addressing the underlying cause.

Mediastinal lymph nodes reside within the mediastinum, the region between the lungs that houses vital structures such as the heart, esophagus, trachea, cardiac nerves, thymus gland, and central chest lymph nodes. When these lymph nodes swell due to malignancy, the leading causes are typically lung cancer and lymphoma. Additionally, enlarged lymph nodes may signal acute lymphoblastic leukemia (ALL). Enlarged lymph nodes can also arise from metastasis (spread) of various cancers, including esophageal, prostate, and gastrointestinal cancers.

Mesenteric lymph nodes commonly enlarge, but this does not always indicate lymphoma involvement. In cases of partial involvement, lymphoma may be detected within the sinuses or the paracortex. Enlarged nodes without tumor involvement may exhibit nonspecific reactive changes, edema, or cavitation. When cavitation occurs, lymph nodes can enlarge significantly (up to 8 cm) due to cystic alterations. In this scenario, the lymph node comprises a thickened capsule and a narrow band of lymphoid tissue encircling clear or turbid fluid, likely lymph. These nodes are located within the mesentery, the membrane connecting the intestines to the posterior abdominal wall.

Cervical lymph nodes, small glands in the neck, may enlarge in response to nearby infections or other health conditions. Treatment for swollen cervical lymph nodes depends on determining the underlying cause. These nodes are positioned in the neck’s front, sides, and back, categorized into anterior and posterior regions. Typically measuring less than 1 centimeter in diameter, larger sizes may indicate infections or health issues such as tumors. The presence of swollen cervical lymph nodes may or may not be apparent to the individual. However, a doctor can identify one or more bumps beneath the skin during a neck examination. Various conditions can lead to swollen cervical lymph nodes, each with its own symptoms. Potential factors contributing to the situation include infections, autoimmune diseases, inflammatory conditions, tumors or cancer, congenital genetic conditions, and medication use or exposure to toxins.

**Fig. 6:**
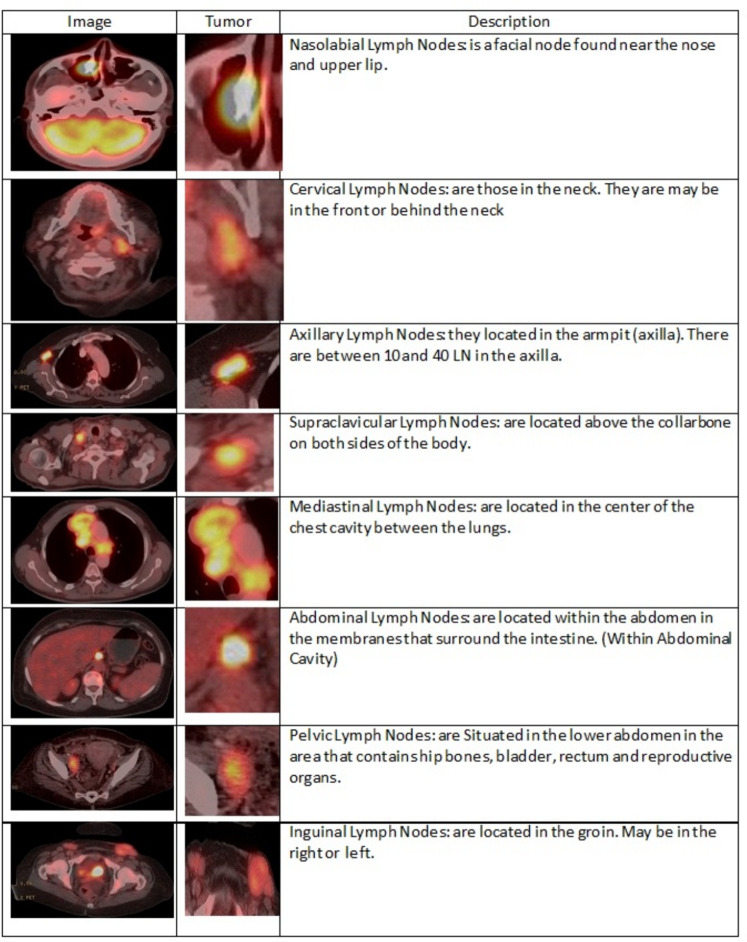
Samples of the lymph nodes.
- Inguinal lymph nodes, found in the groin area, are an integral component of the lymphatic system, just like all other lymph nodes. They in the groin collaborate with the immune system to combat diseases and infections. Swelling in these nodes typically indicates the body’s response to an infection or illness, although it rarely signals cancer. In the inner upper thigh area, superficial inguinal lymph nodes lie near the skin’s surface just below the inguinal ligament. In contrast, the deep inguinal lymph nodes are situated deeper within the body. These lymph nodes are small, oval-shaped, and bean-like, with a typical width of about 1/4 inch. An abnormal inguinal lymph node appears more round than oval and is considered abnormal when its width exceeds 1/2 inch. Enlargement of inguinal lymph nodes may indicate the body’s efforts to combat a disease or infection in the lower body.

The symptoms of swollen lymph nodes will differ depending on the underlying cause. Nevertheless, common indicators of an infection include:

- Pelvic lymph nodes are intricately interconnected through a network of afferent and efferent pelvic lymphatic vessels. These nodes act as vital filtering stations, receiving lymph from pelvic organs via afferent lymphatic vessels and effectively screening for harmful substances such as bacteria, viruses, parasites, and other foreign materials. Situated alongside the internal, external, and common iliac arteries, which supply the lower abdomen and trunk, the pelvic lymph nodes can be affected by retroperitoneal malignancies, commonly affecting the aorta and inferior vena cava, particularly Hodgkin or non-Hodgkin lymphoma, and the lower abdominal structures, including the internal iliac, external iliac, and common iliac lymph nodes.
- Retroperitoneal lymph nodes located in the abdominal region comprise the inferior diaphragmatic nodes and lumbar nodes, which further divide into left lumbar (aortic), intermediate (interaorticovenous), and right lumbar (caval) nodes. Retroperitoneal malignancies commonly involve the aorta and inferior vena cava, particularly Hodgkin or non-Hodgkin lymphoma. Hodgkin’s lymphoma often involves contiguous spread to the spleen and retroperitoneum. Effective treatment for retroperitoneal malignant lymphoma typically involves chemotherapy and radiotherapy, with surgical interventions being cautioned against. Located behind the abdominal cavity, these nodes necessitate careful consideration for medical procedures. Inguinal lymph nodes, on the other hand, are situated in the groin area. Figure 6 shows the examples and their description.

We have collected a wide range of cases, including primary and secondary lymph node tumors that have spread from other lymph nodes. As a team, we’ve amassed a substantial collection of medical images showcasing lymph node tumors across various body regions. A noteworthy advancement in our work is the development of fusion images that merge PET-CT and CT images. This innovation produces unique images that aid in the improved diagnosis of lymph node tumors and other tumors requiring detailed examination.

### The performance measures

We employed four distinct metrics to assess the effectiveness of the proposed system: precision, recall, Dice similarity coefficient (DSC), and mean average precision (mAP). They are among the key evaluation metrics used as defined by Eqs. (1–5)^52^.

1 $$\begin{aligned} & recall=\frac{TP}{TP+FN}, \end{aligned}$$

2 $$\begin{aligned} & DSC=\frac{2\times TP}{2\times TP +FP+FN}, \end{aligned}$$

3 $$\begin{aligned} & Precision =\frac{TP}{TP+FP}, \end{aligned}$$

4 $$\begin{aligned} & IoU = \frac{Area of intersection}{Area of union} \end{aligned}$$

5 $$\begin{aligned} & mAP =\frac{1}{n}\sum {k=1}^{k=n}{AP(IoU)}_{k} \end{aligned}$$

In this context, *TP*, *FP*, *TN*, and *FN* denote true positives, false positives, true negatives, and false negatives, respectively. TP indicates that the object detection model correctly identified the object type at the correct location. FP means the model has identified an object that is not there or assigned the wrong label. FN means that the model has not identified an object that it should have. FN occurs when the model fails to detect or misses actual objects in the image.

Object detection is a widely recognized challenge in computer vision, involving the identification and categorization of objects in images. mAP is a standard metric employed to assess the precision of an object detection model^53^. In object detection tasks, precision is determined with respect to threshold^54^. The precision value varies with the IoU threshold. IoU is a straightforward measure that indicates the degree of overlap between the predicted and ground-truth bounding boxes. To further dissect the term, ”intersection” refers to the shared area between the predicted and labeled initial regions. At the same time, ”union” encompasses the total area covered by the predicted and ground truth bounding boxes.

For each class k, the mAP is computed across various IoU thresholds, and the overall test mAP is computed by averaging the mAPs for each class. AP_k_ is the AP for class k, where n represents the total number of classes. Precision evaluates the accuracy of predictions, while recall assesses the quantity of predictions that match the ground truth. The DSC, calculated as the harmonic mean of precision and recall, evaluates a model’s performance by accounting for false positives and false negatives^55^.

## The experimental results

This section offers a detailed examination of the exclusive dataset and describes the performance metrics. Besides, the outcomes achieved by implementing the proposed model are presented.

### The results

Python versions 3.7 and 3.12 were used to implement the suggested framework, which was run locally and on Google Colab. The local computer was used for testing, validation, and performance assessment, while Google Colab was primarily used for model training due to its GPU acceleration.

TensorFlow 2.4 is the primary framework for developing deep learning models. Image processing operations such as scaling, normalization, and augmentation were performed using OpenCV. Format conversion, preprocessing, and dataset annotations were done using Roboflow. The local experimental setup consisted of an Intel Core i5 processor running at 2.4 GHz, 8 GB of RAM, and an NVIDIA GPU with 1 GB of VRAM. We applied all seven built-in models: YOLOv7, YOLOv8 (l, s), YOLOv9, YOLOv10, YOLOv11, YOLOv12, and YOLO-Nas to compare and select the model that improved performance in detecting the 13 lymph node classes on the established dataset. Each image may contain multiple classes.

For object detection, the YOLO-based models were implemented and trained on the prepared dataset. The input images were resized to a fixed resolution of $640\times 640$. The models were trained using a batch size of 16 and a stride of 32 for 200 epochs. The learning rate was initialized at $5 \times 10^{-3}$. Adamax optimizer was applied, Momentum was 0.937, and weight decay was 0.0005

During training, an Intersection over Union (IoU) threshold of 0.5 was used to determine true positive detections. Performance was evaluated using mean Average Precision (mAP), computed at an IoU threshold of 0.5 (mAP@0.5).

At first, we applied the standard YOLOv8 on our dataset and recorded the results as shown in Table 5.

**Table 5:** Standard YOLOv8n results of P, R, mAp50, and DSC for 13 predicted classes of 15 annotated classes of the LYN23 dataset.

Classes	Precision	Recall	mAP50	DSC
Abdominal	0.88	0.80	0.87	0.90
Cervical	0.87	1	1	0.88
It-subclavicular	0.66	0.48	0.33	0.74
Lt-inguinal	0.47	0.61	0.63	0.70
Mediastinal	0.83	0.70	0.79	0.82
Normal	0.85	1	0.98	0.89
Pelvis	0.51	0.88	0.83	0.91
Rt-cervical	0.78	0.67	0.77	0.90
Rt-subclavicular	0.34	0.52	0.56	0.79
Rt-axillary	0.77	0.71	0.81	0.88
Rt-inguinal	0.68	0.43	0.66	0.87
lt-axillary	0.84	0.87	0.86	0.86
lt-cervical	0.65	0.46	0.63	0.79
Total	0.65	0.66	0.81	0.66

From Table 5, we can notice that the standard Yolov8n model achieved 65%, 66%, 81%, and 66% for P, R, mAp50, and DSC repectively. It detected only 13 classes and could not predict noise and retroperitoneal classes. On the other hand, it achieved the best results in detecting abdominal class, outperforming other classes. The worst results are achieved for detecting Rt–subclavicular, with Precision, recall, and mAP50 of 34%, 52%, and 56%, respectively.

**Fig. 7:**
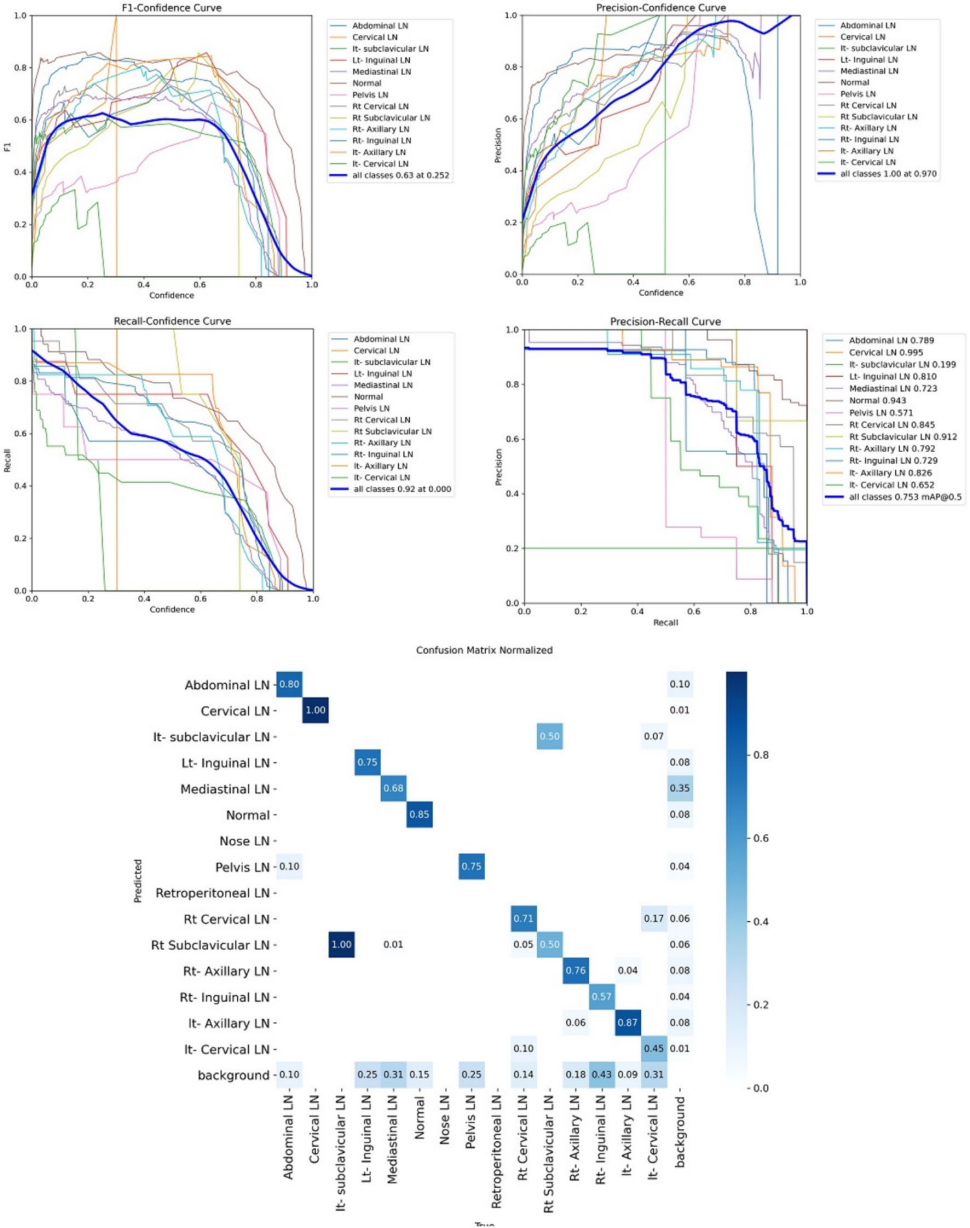
From top right to bottom left, the curves are the F1, P, R, and PR curves and the Confusion Matrix in the bottom of only 13 classes that were predicted of the LYE23 dataset using standard YOLOv8n.

Figure 7 shows the various curves of F1, P, R, and PR that achieved max points at 63%, 100%, 75%, and 92%, respectively. In addition to the confusion matrix of the standard YOLOv8n. Only one class achieved 100%, which is cervical. The second order is for the it-axillary, which achieved 87%. Then the Normal class, which equals 85%.

Second, we changed the kernel to be $7\times 7$ and we recorded the following results shown in Table 6.

**Table 6:** The modified YOLOv8 results of P, R, mAp50, and DSC due to increasing kernel to $7\times 7$ for 13 predicted classes of 15 annotated classes of the LYN23 dataset.

Classes	Precision	Recall	mAP50	DSC
Abdominal	0.96	0.74	0.90	0.84
Cervical	1	0	0.02	0
It-subclavicular	0.32	0.5	0.6	0.39
Lt-inguinal	0.69	0.63	0.63	0.66
Mediastinal	0.89	0.56	0.81	0.69
Normal	0.96	0.72	0.97	0.82
Pelvis	0.54	0.88	0.71	0.67
Rt-cervical	0.85	0.81	0.92	0.83
Rt-subclavicular	0.34	0.52	0.56	0.79
Rt-axillary	0.83	0.57	0.88	0.68
Rt-inguinal	0.82	0.43	0.75	0.57
Lt-axillary	0.83	0.84	0.89	0.84
Lt-cervical	1	0.41	0.72	0.58
Total	0.82	0.62	0.74	0.71

From Table 6, we can notice that by increasing the kernel, the modified model achieved 82%, 62%, 74%, and 71% for P, R, mAP50, and DSC, respectively. It also detected only 13 classes from 15 annotated classes.

**Fig. 8:**
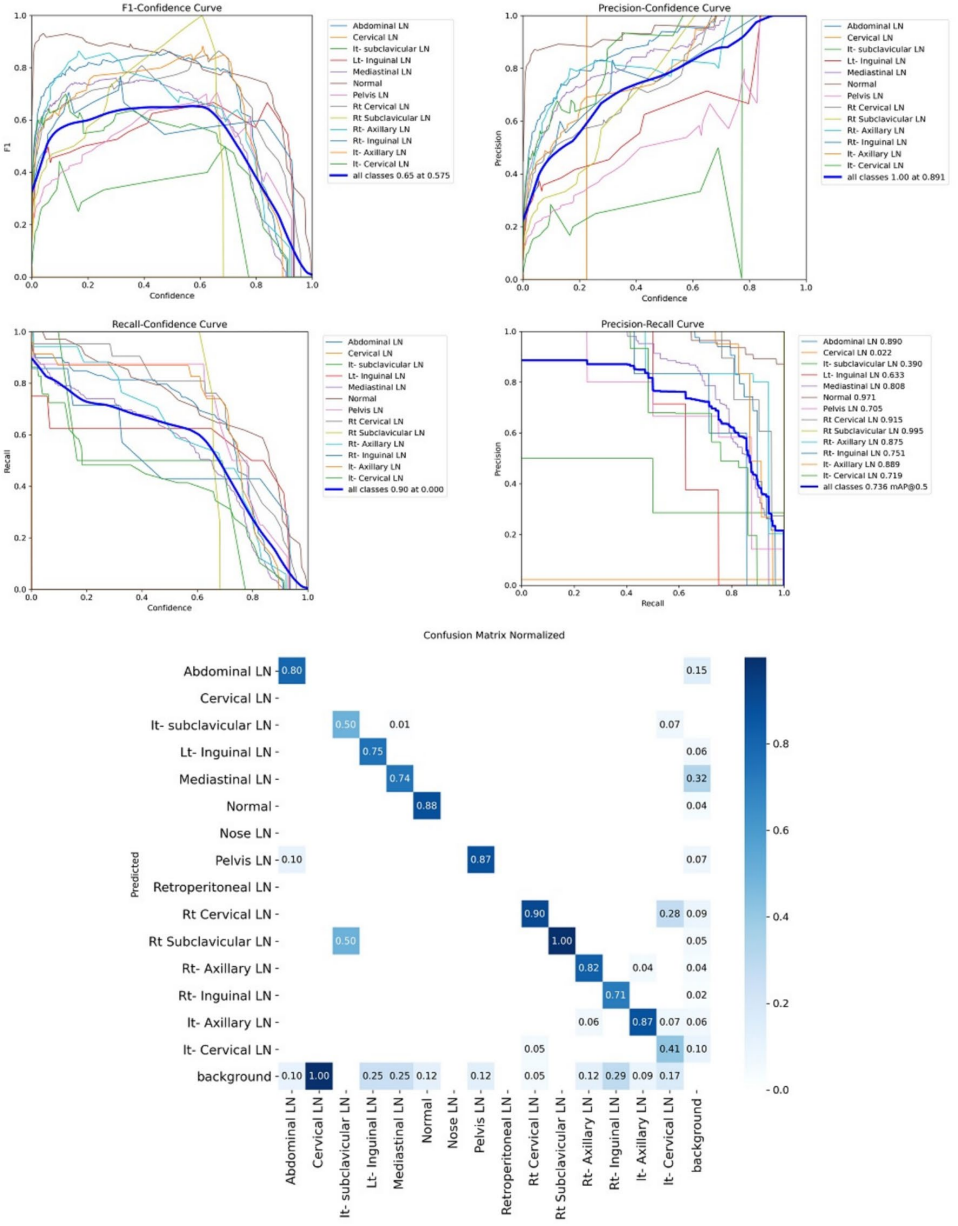
From top right to bottom left, the curves are the F1, P, R, and PR curves and the Confusion Matrix in the bottom of only 13 classes that were predicted of the LYE23 dataset using modified YOLOv8 based on increasing kernel to $7\times 7$.

Figure 8 shows the four curves and the confusion matrix after increasing the kernel to $7\times 7$. The figure shows the curves for F1, P, R, and PR, which achieved max points at 65%, 100%, 74%, and 90%, respectively. The confusion matrix shows that the best performing classes are Rt Subclavicular, Rt Cervical, Normal, Pelvis, and Lt Axillary, which achieved 100%, 90%, 88%, 87%, and 87%, respectively. Third, we recorded the results based on changing various parameters as shown in the Table 4.

**Table 7:** The modified YOLOv8 results of P, R, mAp50, and DSC due to changing hyperparameters for 13 predicted classes of 15 annotated classes of the LYN23 dataset.

Classes	Precision	Recall	mAP50	DSC
Abdominal	0.77	0.85	0.81	0.68
Cervical	0.4	1	1	0.57
It-subclavicular	0.11	0.5	0.2	0.2
Lt-inguinal	0.44	0.69	0.63	0.54
Mediastinal	0.75	0.70	0.77	0.73
Normal	0.89	0.91	0.97	0.90
Pelvis	0.40	0.88	0.87	0.55
Rt-cervical	0.56	0.81	0.82	0.66
Rt-subclavicular	0.63	0.85	0.75	0.75
Rt-axillary	0.78	0.85	0.88	0.82
Rt-inguinal	0.74	0.82	0.85	0.78
lt-axillary	0.61	0.91	0.82	0.74
lt-cervical	0.60	0.52	0.60	0.56
Total	0.59	0.79	0.77	0.68

Table 7 shows that the modified model with changing the various hyperparameters achieved 59%, 79%, 77%, and 68% for P, R, mAP05, and DSC, respectively.

**Fig. 9:**
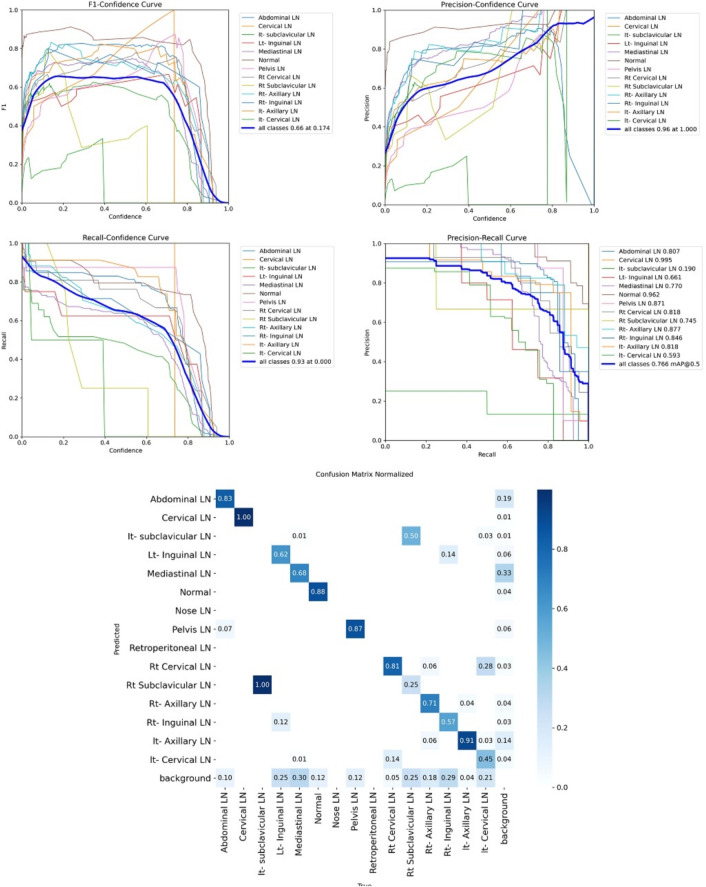
From top right to bottom left, the curves are the F1, P, R, and PR curves and the Confusion Matrix in the bottom of only 13 classes that were predicted of the LYE23 dataset using modified YOLOv8 based on hyperparameters.

Figure 9 shows the four curves and the confusion matrix after changing hyperparameters. The figure shows the curves for F1, P, R, and PR, which achieved max points at 66%, 96%, 77%, and 93%, respectively. The confusion matrix shows that the best performing classes are Cervical, Lt Axillary, Normal, Pelvis, Abdominal, and Right Cervical that achieved 100%, 91%, 88%, 87%, 83% and 81% respectively. Fourth, We added extra Conv layers and recorded the results as shown in Table 8.

**Table 8:** The modified YOLOv8 results of P, R, mAp50, and DSC due to adding extra conv layers for 13 predicted classes of 15 annotated classes of the LYN23 dataset.

Classes	Precision	Recall	mAP50	DSC
Abdominal	0.81	0.78	0.80	0.8
Cervical	0.4	1	1	0.57
It-subclavicular	0.31	0.5	0.3	0.39
Lt-inguinal	0.58	0.75	0.74	0.65
Mediastinal	0.83	0.70	0.8	0.76
Normal	0.94	0.97	0.98	0.96
Pelvis	0.31	0.88	0.72	0.46
Rt-cervical	0.67	0.91	0.86	0.77
Rt-subclavicular	0.44	0.5	0.57	0.47
Rt-axillary	0.72	0.71	0.79	0.72
Rt-inguinal	0.71	0.57	0.75	0.63
lt-axillary	0.65	0.91	0.90	0.76
lt-cervical	0.81	0.43	0.59	0.56
Total	0.63	0.74	0.75	0.68

Table 8 shows that the modified model with adding extra conv layers achieved 63%, 74%, 75%, and 68% for P, R, mAP05, and DSC, respectively.

**Fig. 10:**
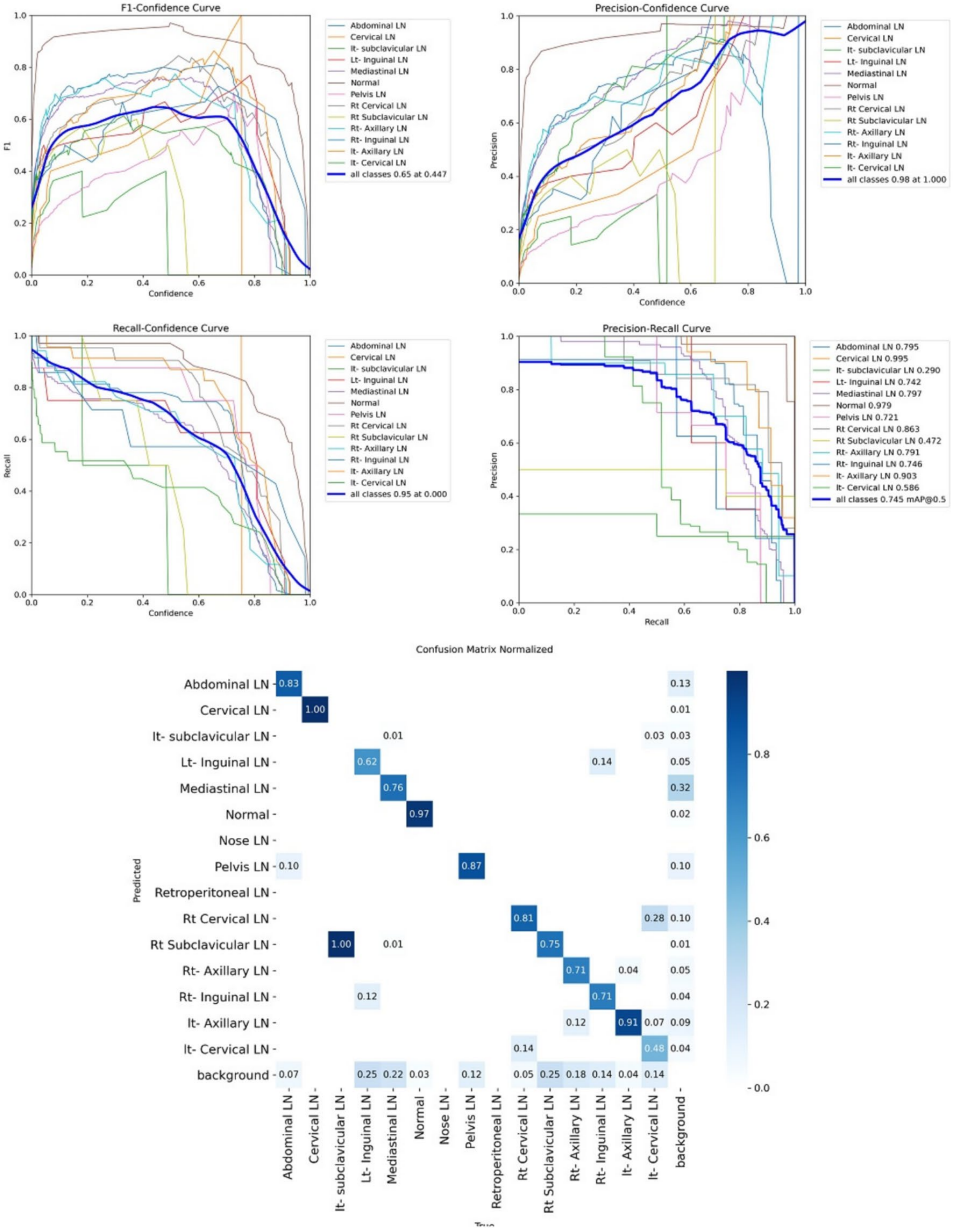
From top right to bottom left, the curves are the F1, P, R, and PR curves and the Confusion Matrix in the bottom of only 13 classes that were predicted of the LYE23 dataset using modified YOLOv8 based on adding extra conv layers.

Figure 10 shows the four curves and the confusion matrix after adding extra Conv layers. The figure shows the curves for F1, P, R, and PR, which achieved max points at 65%, 98%, 75%, and 95%, respectively. The confusion matrix shows that the best-performing classes are Cervical, Normal, Lt Axillary, Pelvis, Abdominal, and Rt Cervical, which achieved 100%, 97%, 91%, 87%, 83%, and 81%, respectively. Fifth, Table 9 shows the precision, recall, mAP50, and DSC results after applying the modified YOLOv8 after adding P6 in addition to the previous four changes. The model accurately predicted the Rt-subclavicular class, with precision, recall, mAP50, and DSC of 100%, 96%, 100%, and 76%, respectively. Lt-axillary ranked second, with 93%, 93%, 98% , and 0.88 for the performance above. The results for the abdominal and mediastinal areas are nearly similar. They are 86%, 86%, 90%, 91% for abdominal resection, recall, mAP50, and DSC, respectively, and 90%, 83%, 89% , and 95%for mediastinal. The model achieved the worst precision in the cervical class. The class which was difficult in annotation, as the left and right cervical lymph nodes were overlapped. On the other hand, the model achieved 100% for recall and mAP50 for the same class. For the normal class, the modified model achieved 92%, 100%, 99%, and 93% across the four measures, respectively. For the pelvic class, the model achieved only 54% precision but higher values for recall and mAP50, at 92% and 87%, respectively. It achieved a moderate DSC value of 73%. In conclusion, the model achieved 75%, 83%, 85%, and 77% for precision, recall, mAP50, and DSC across all classes, respectively. The increase in mAP to 85% is attributed to the addition of deeper feature representation (P6) and increased channel capacity, which enhances detection of large objects. However, this comes at the cost of higher computational complexity. The proposed model achieved a precision of 75% and a recall of 83%, indicating that it is more effective at detecting objects than avoiding false positives. The high recall demonstrates strong detection capability, while the slightly lower precision suggests some false-positive predictions.

**Table 9:** The modified YOLOv8 results of precision, recall, mAp50, and DSC based on adding P6 for only 13 classes of LYN23 dataset were finally detected by the proposed model.

Classes	Precision	Recall	mAP50	DSC
Abdominal	0.86	0.85	0.9	0.91
Cervical	0.37	1	1	0.89
It- subclavicular	0.62	0.82	0.66	0.75
Lt- Inguinal	0.66	0.73	0.73	0.80
Mediastinal	0.9	0.83	0.89	0.95
Normal	0.92	1	0.99	0.93
Pelvic	0.54	0.92	0.87	0.70
Rt Cervical	0.73	1	0.94	0.85
Rt Subclavicular	1	0.96	1	0.75
Rt- Axillary	0.8	0.68	0.76	0.78
Rt- Inguinal	0.77	0.57	0.64	0.80
lt- Axillary	0.93	0.93	0.98	0.88
lt- Cervical	0.86	0.63	0.76	0.92
Total	0.75	0.83	0.85	0.77

**Fig. 11:**
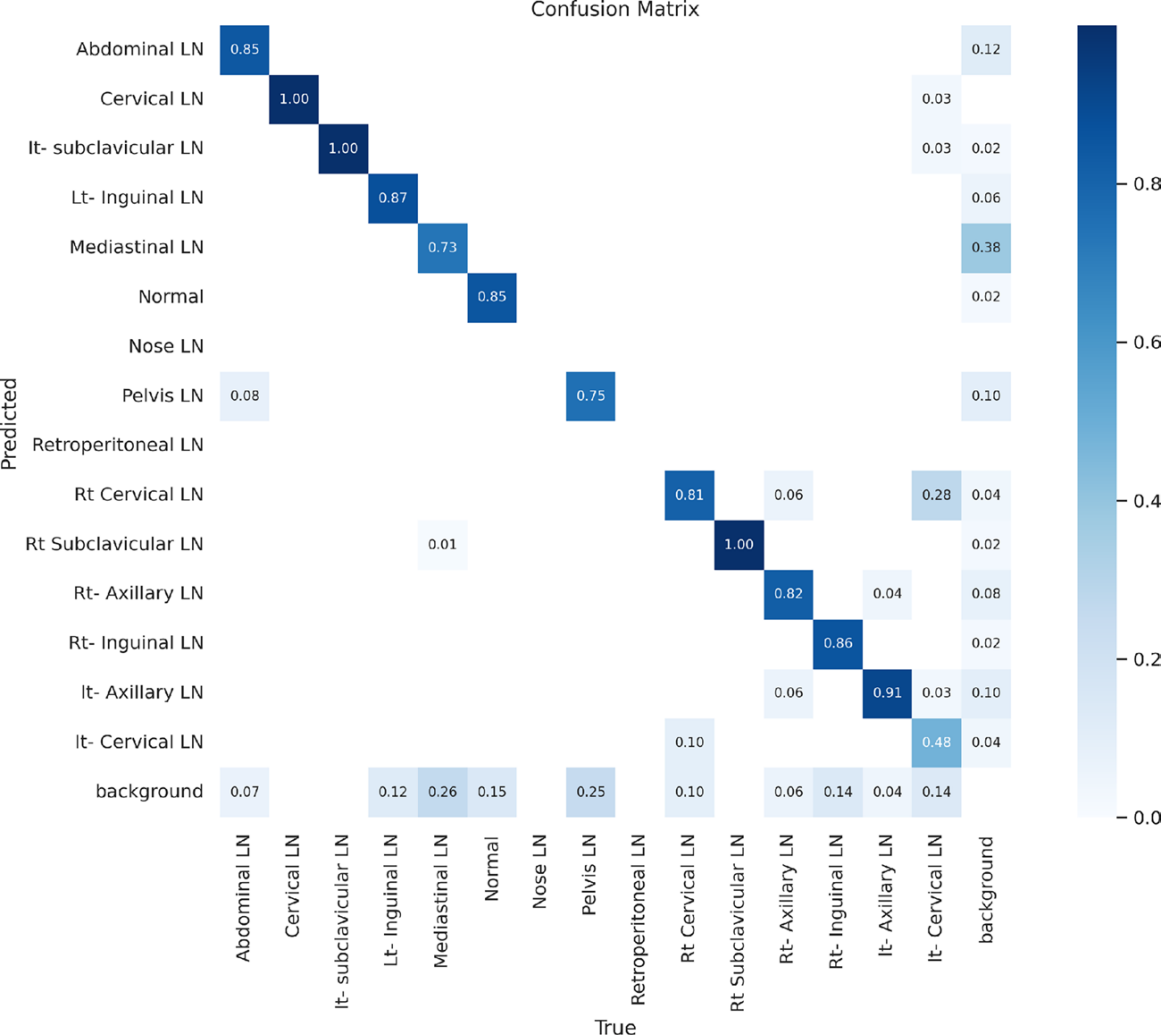
The confusion matrix of the actual labeled and the predicted classes of the modified YOLOv8. All annotated 15 classes of the dataset appeared in addition to the ignored background, but only 13 classes were finally detected by the proposed model.

Figure 11 presents the confusion matrix for all classes. It presents the prediction and actual labeled classes. We notice that the most accurately predicted classes, such as the true-labeled ones, are cervical, Rt-cervical, and Rt-subclavicular. The normal class achieved 92%, 100%, and 99%, respectively. Lt-axillary and pelvic classes ranked third, achieving 93% and 92%, respectively. Abdominal and mediastinal are equal to 88% and 84%, respectively. It-subclavicular and Lt-inguinal are both 75%. Rt-axillary and lt-cervical are equal to 68% and 60%, respectively. Finally, the Rt-inguinal class ranks last, with a frequency of 57%.

**Fig. 12:**
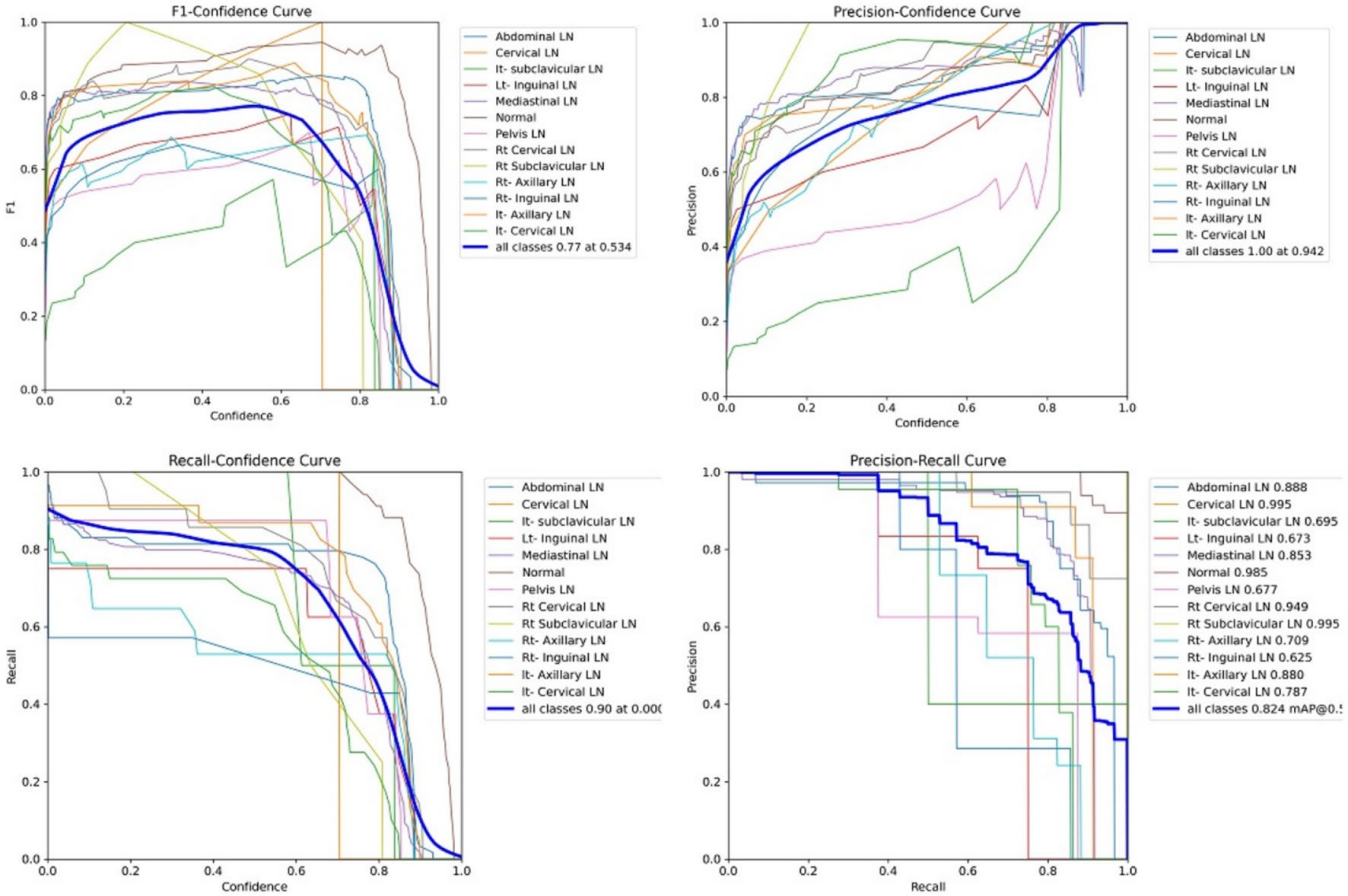
From top right to bottom left, the curves are the F1, P, R, and PR curves for only 13 detected classes of 15 annotated classes of the LYE23 dataset using the modified YOLOv8.

**Fig. 13:**
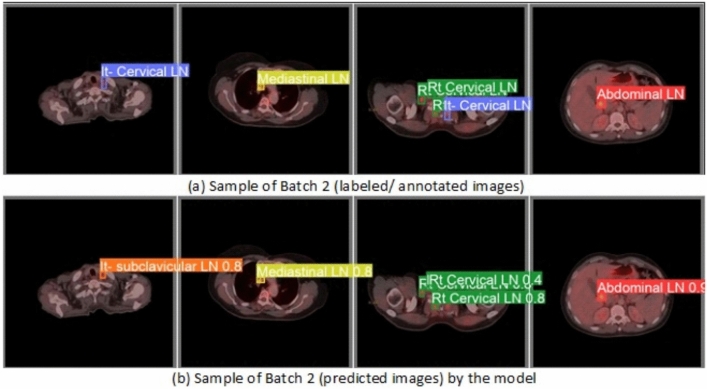
A sample of batch 3 containing (**a**) the labeled or annotated images for three images and (**b**) the predicted same three images as output from the modified model for only 13 classes of the LYE23 dataset..

**Fig. 14:**
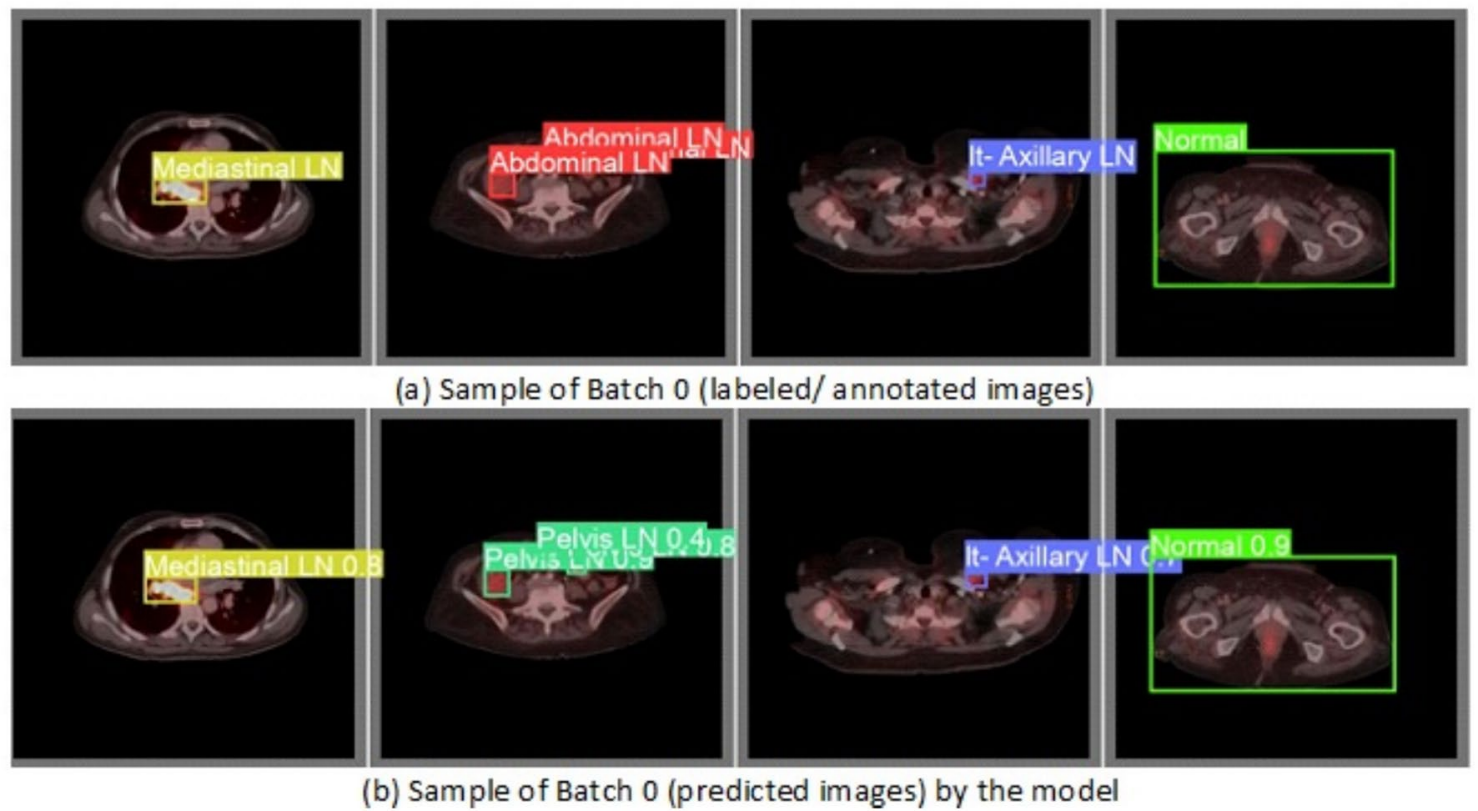
A sample of batch 0 containing (**a**) the labeled or annotated images for three images and (**b**) the predicted same three images as output from the modified model for only the 13 predicted classes from 15 annotated classes of LYE23 dataset.

**Fig. 15:**
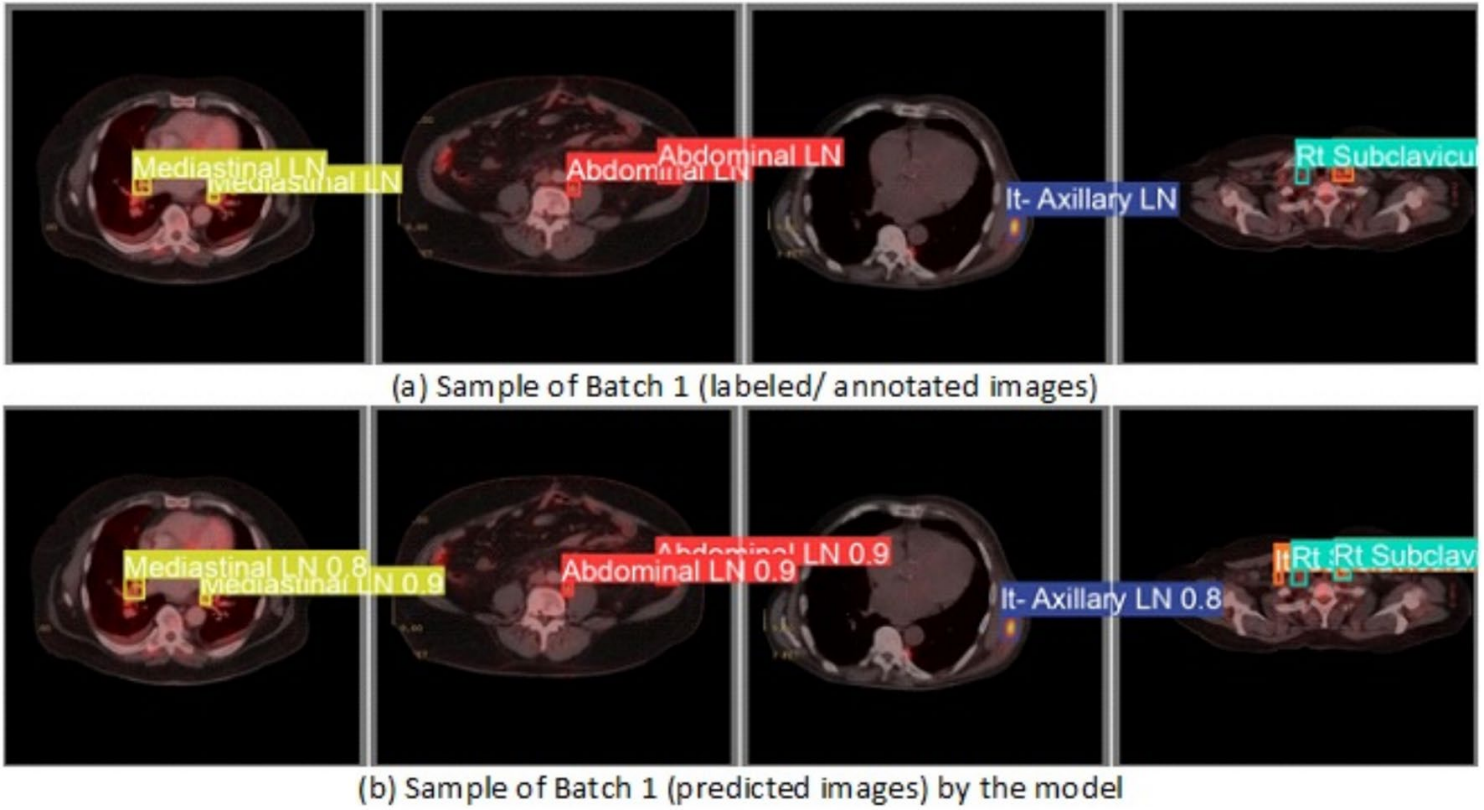
A sample of batch 1 containing (**a**) the labeled or annotated images for three images and (**b**) the predicted same three images as output from the modified model for only the 13 predicted classes from 15 annotated classes of the LYE23 dataset.

From the CM shown in Fig. 11, we observe that the true labels predicted accurately at 100% are cervical, Rt-cervical, and Rt-subclavicular. Rt-axillary and pelvis achieved 93% and 92%, respectively. Abdominal, normal, and mediastinal achieved 88%, 85%, and 84%, respectively. It-subclavicular and Lt-inguinal are the same because they achieved 75%. Rt-axillary and lt-cervical achieved 68% and 60%, respectively. Finally, Rt-inguinal achieved 57%. Figure 12 shows the DSC, precision (P), and recall (R) results, which equal 77%, 100%, and 90%, respectively. It presents the DSC, P, R, and PR curves. Figures 13, 14, and 15 show examples of PET/CT images annotated and predicted. By increasing the batch size from 16 to 64 and running for 55 epochs, F1, P, R, and PR are 72%, 100%, 93%, and 75%, respectively

As we noticed that the modified Yolov8 detected only 13 classes and did not detect the nose and retroperitoneal classes. We can conclude that 15 classes were annotated in the dataset, but only 13 classes were finally detected by the proposed model. Table 10 shows the effect of each important modification separately on our dataset.

**Table 10:** The various modifications aspects on Yolov8n.

Modification	Precision	Recall	mAP50	DSC	GFLOP
YOLOv8n	0.65	0.66	0.81	0.66	8.1
Kernel $7\times 7$	0.82	0.62	0.74	0.71	12.7
Hyperparameters	0.59	0.79	0.77	0.68	12.7
Extra CONVs	0.63	0.74	0.75	0.68	23.7
Full Model with P6	0.75	0.83	0.85	0.77	44.1

From Table 10, the baseline YOLOv8 achieved 0.65, 0.66, 0.81, 0.66, and 8.1 for P, R, mAP05, and GFLOPs, respectively. It is balanced but not outstanding in any metric, with very low computation. The base model with Kernel 7$\times$7 achieved 0.82, 0.62, 0.74, 0.71, and 12.7 for P, R, mAP50, DSC, and GFLOPs. It achieved high precision, meaning fewer FP but low R misses more true objects missed. The model with hyperparameter modifications achieved 0.59, 0.79, 0.77, 0.68, and 12.7 for P, R, mAP50, DSC, and GFLOPs. It detects most objects, but has more FP. The model with Extra Convs layers achieved 0.63, 0.74, 0.75, 0.68, and 23.7 for P, R, mAP50, DSC, and GFLOPs. It improved R with a mild decrease in mAP and DSC, while the computations nearly doubled compared to the baseline Yolov8n model. The full model with p6 achieved 0.75, 0.83, 0.85, 0.77, and 44.1 for P, R, mAP50, DSC, and GFLOPs. The computations are very expensive,  5$\times$ baseline. But it achieved the best accuracy.

We split the data again into 5 folds and recorded the results as shown in Table 11.

**Fig. 16:**
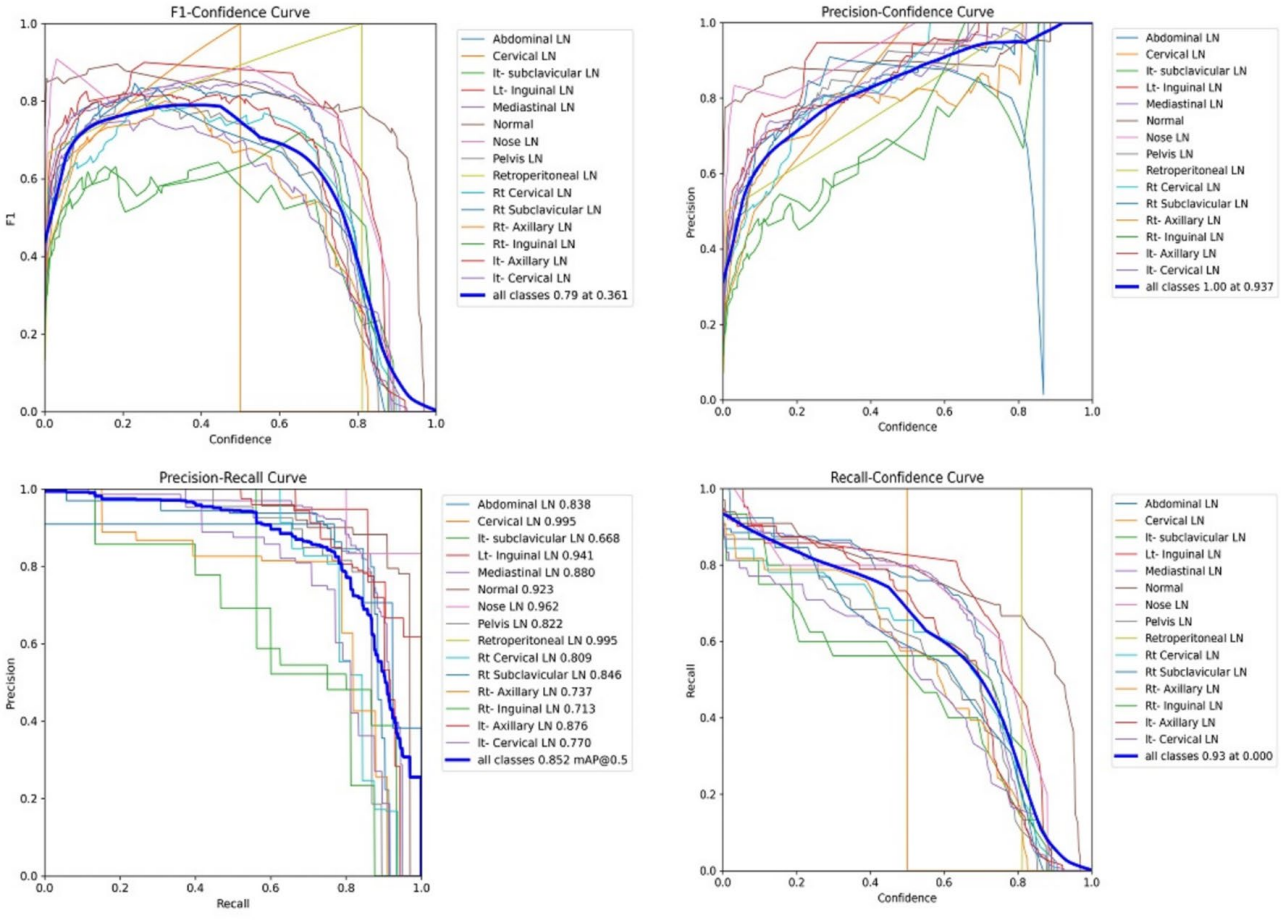
The plotted curves of the modified YOLOv8 model are based on 5-folds cross-validation (Fold 4). From top right to bottom left, the curves are the F1, P, PR, and R curves for all classes of the LYE23 dataset using modified YOLOv8.

**Fig. 17:**
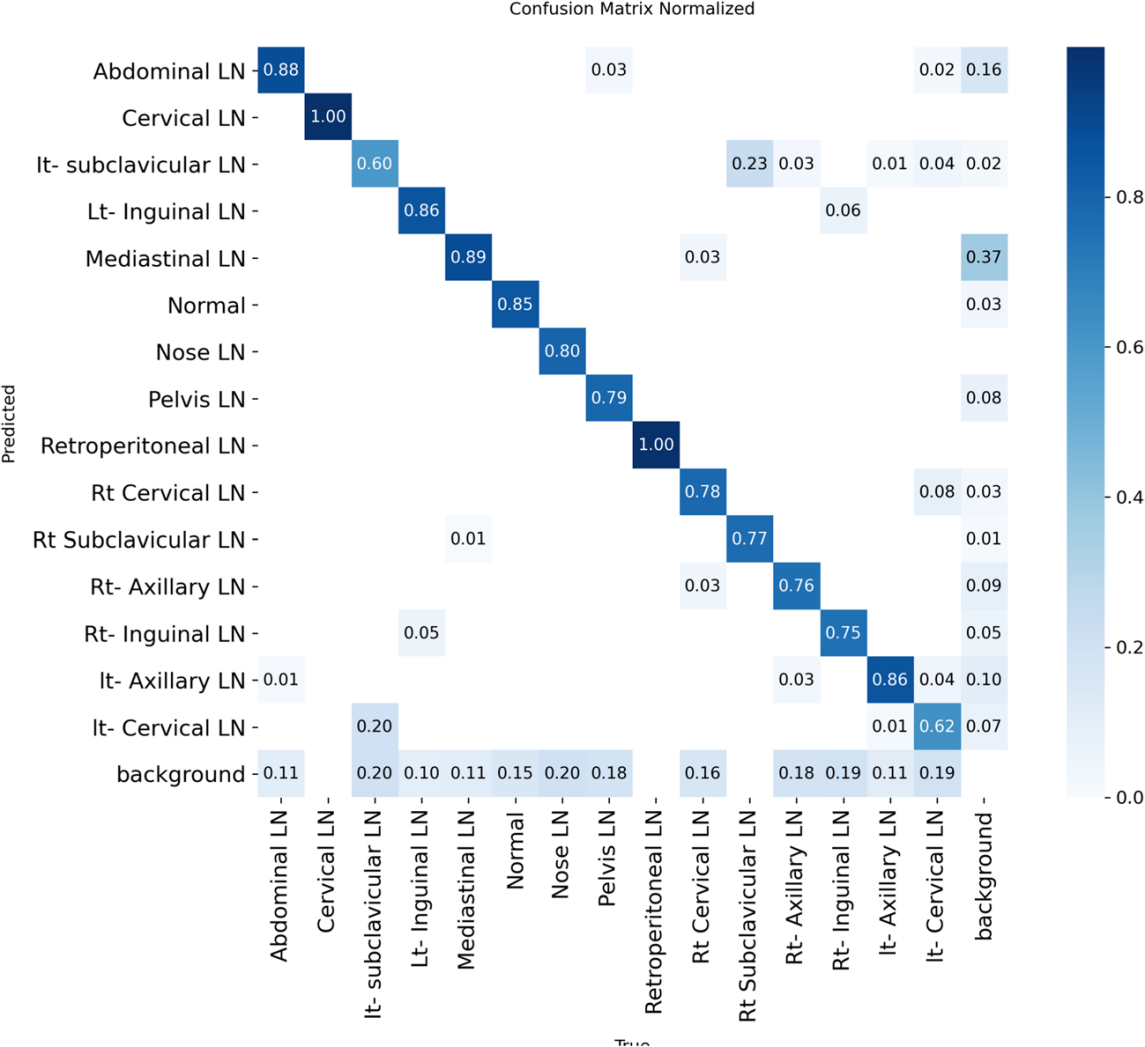
The confusion matrix for all classes of the LYE23 dataset using the modified YOLOv8 is based on fold 4.

From the previous table, we can see that fold 4 yields the best results, detecting all 15 classes, including nose and retroperitoneal. The modified model with fold 4 provides 81%, 79%, 85%, and 80% for P, R, mAP50, and DSC, respectively. The modified model by using fold 2 and 3 could not detect the retroperitoneal class and gave the worst results in detecting the nose class in fold 3. Moreover, the modified model could not detect the cervical class by using fold 5 and gave 41%, 70%, 69%, and 52% for P, R. mAP, and DSC, respectively. Figure 16 shows the various curves of the resulting values of the modified YOLOv8 based on Fold4. Figure 17 shows the confusion matrix for all classes of the LYE23 dataset using the modified YOLOv8 based on fold 4.

**Table 11:** P, R, mAP50, and DSC of all classes by using 5-folds cross-validations of the modified Yolov8.

	Fold1				Fold2				Fold3				Fold4				Fold5			
**class**	**P**	**R**	**mAP**	**DSC**	**P**	**R**	**mAP**	**DSC**	**P**	**R**	**mAP**	**DSC**	**P**	**R**	**mAP**	**DSC**	**P**	**R**	**mAP**	**DSC**
Abdominal	0.82	0.81	0.84	0.80	0.90	0.72	0.84	0.78	0.93	0.72	0.86	0.82	0.83	0.85	0.84	0.83	0.79	0.86	0.86	0.82
Cervical	0.29	0.5	0.60	0.05	0.65	0.50	0.70	0	0.74	0.5	0.5	0.64	0.83	1	1	0.83	-	-	-	-
It- subclavicular	0.77	0.65	0.75	0.65	0.66	0.24	0.52	0.56	1	0.53	0.79	0.82	0.60	0.6	0.67	0.60	0.45	0.53	0.43	0.49
Lt- Inguinal	0.86	0.65	0.75	0.70	0.79	0.76	0.95	0.75	0.59	0.58	0.63	0.60	0.95	0.84	0.94	0.95	0.78	1	0.98	0.88
Mediastinal	0.81	0.84	0.85	0.80	0.88	0.75	0.83	0.88	0.80	0.88	0.80	0.83	0.84	0.88	0.84	0.86	0.80	0.83	0.85	0.82
Normal	0.86	0.83	0.91	0.86	0.91	0.67	0.91	0.84	0.80	0.81	0.86	0.80	0.87	0.83	0.92	0.85	1	0.81	0.90	0.89
Nose	1	0.87	0.86	0.86	0.86	0.62	1	0.80	0.59	0.25	0.32	0.38	0.91	0.80	0.96	0.89	0.41	0.70	0.69	0.52
Pelvis	0.82	0.83	0.88	0.83	0.75	0.74	0.84	0.74	0.74	0.70	0.78	0.72	0.87	0.68	0.82	0.77	0.71	0.81	0.79	0.76
retroperitoneal	0.7	1	1	0	-	-	-	-	-	-	-	-	0.72	1	1	0.78	0.62	1	1	0.77
Rt Cervical	0.83	0.71	0.84	0.76	0.82	0.64	0.77	0.67	0.78	0.70	0.78	0.77	0.82	0.75	0.81	0.81	0.70	0.77	0.76	0.73
Rt Subclavicular	0.65	0.70	0.77	0.65	0.67	0.73	0.78	0.71	0.66	0.73	0.71	0.70	0.90	0.67	0.85	0.77	0.85	0.64	0.83	0.73
Rt- Axillary	0.87	0.85	0.93	0.85	0.90	0.77	0.85	0.81	0.78	0.81	0.80	0.78	0.81	0.74	0.77	0.78	0.78	0.91	0.86	0.84
Rt- Inguinal	0.90	0.73	0.92	0.83	0.86	0.70	0.87	0.82	0.70	0.74	0.75	0.71	0.63	0.56	0.71	0.60	0.71	0.83	0.88	0.77
lt- Axillary	0.84	0.81	0.86	0.84	0.87	0.75	0.85	0.80	0.76	0.83	0.80	0.78	0.81	0.81	0.88	0.81	0.78	0.85	0.78	0.81
lt- Cervical	0.75	0.81	0.84	0.78	0.80	0.56	0.74	0.63	0.55	0.43	0.57	0.50	0.81	0.67	0.77	0.78	0.78	0.82	0.82	0.80
Total	0.79	0.78	0.85	0.78	0.79	0.69	0.80	0.78	0.75	0.65	0.72	0.68	0.81	0.79	0.85	0.80	0.73	0.81	0.82	0.77

**Table 12:** The Comparison between 80/10/10 and 5-Folds CV Splitting based on the validation results.

Aspect of comparison	80/10/10	Notes on 80/10/10	5-Folds CV	Notes on 5-Folds CV
No.of Predicted Classes	13	–	15	in average results, all 15 classes were detected
No.of Unpredicted Classes	2	Nose and Retroperitoneal	2	Cervical in fold 5 and Retroperitoneal in folds 2,3
P	0.75	–	0.78	Average P
R	0.83	–	0.75	Average R
mAP	0.85	–	0.81	Average mAP
DSC	0.77	–	0.76	Average DSC

Although the 80/10/10 split achieved a higher validation result as shown in Table 12, the 5-fold cross-validation provides a more reliable estimate of model performance due to reduced variance across multiple splits.

We compared the modified YOLOv8 with YOLOv7, YOLOv8, and YOLO-Nas. For YOLOv8, F1, P, R, PR are 74%, 100%, 92%, and 84%, respectively as maximum points shown in Fig. 7. The difference is 3% in F1 for the modified YOLOv8 with 200 epochs and a batch size of 16. The two models are equal in PR at 84%. However, R in the modified YOLOv8 is 90%, while it is 92% in YOLOv8.

We compared the modified YOLOv8 with YOLOv9, YOLOv10, YOLOv11, and YOLOv12. For example, but not limited to, as shown in Table 14. In YOLOv11, the cervical class had a P of 49%, while it is 37% in the modified model. For RT-cervical, P was 76% in YOLOv11, while the modified model recorded 73%. Apart from that, all results are almost identical across most categories, except for a slight increase in some categories favoring the modified model.

**Fig. 18:**
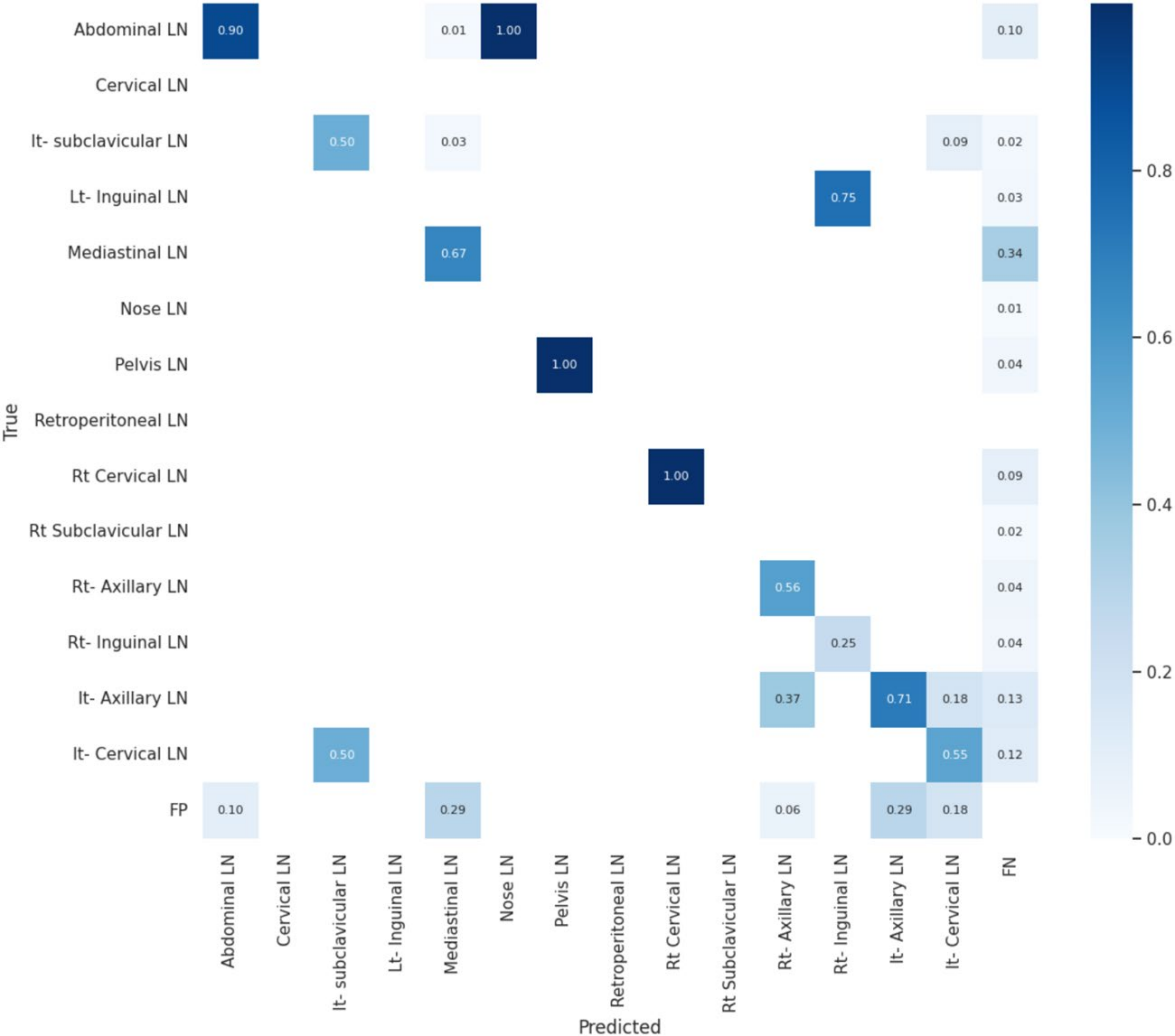
The confusion matrix on YOLO-Nas.

**Fig. 19:**
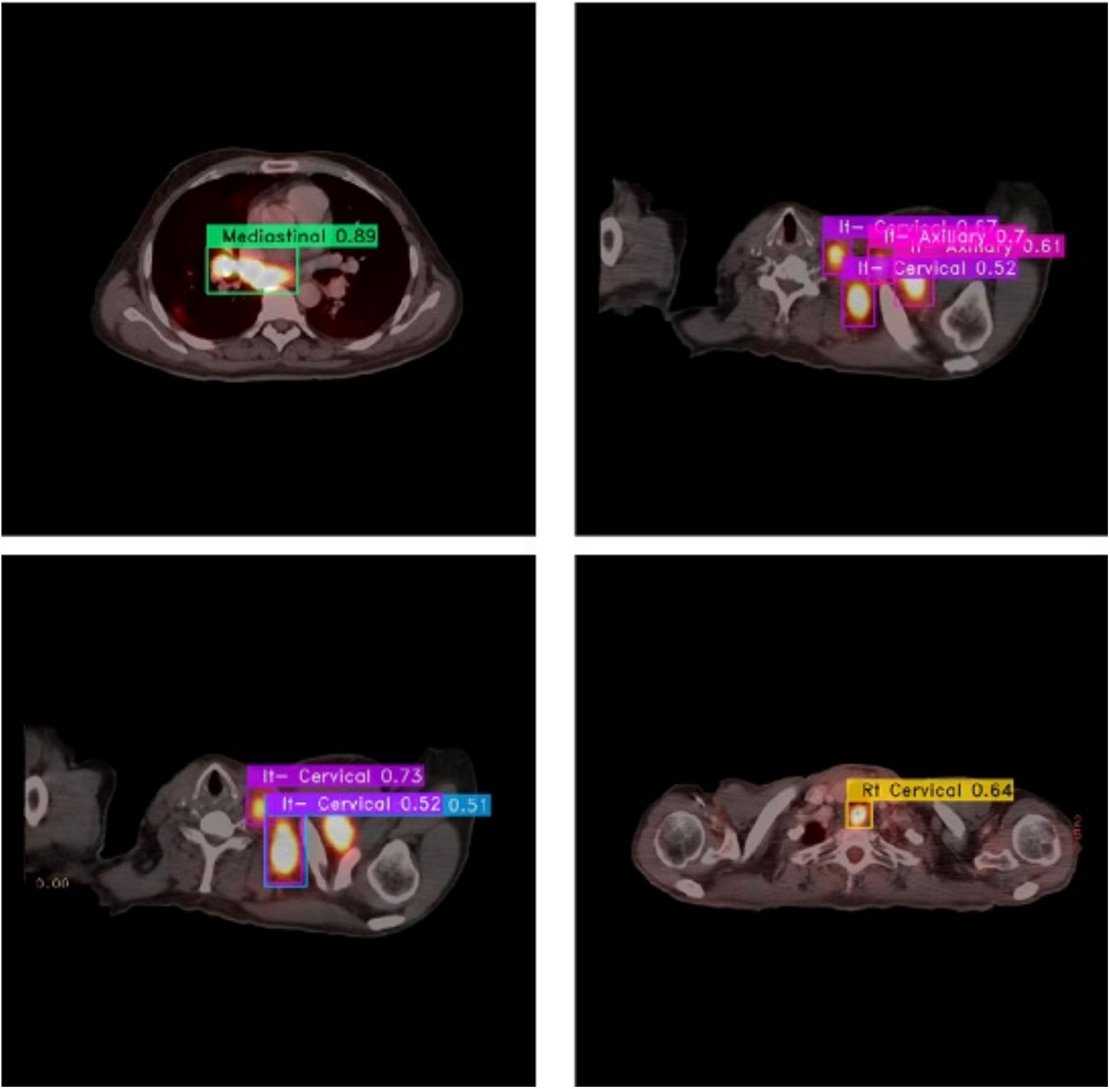
Sample of the detected images from the YOLO-Nas model.

For Yolo-Nas, it takes a long time to train on 50 epochs. We found that loss cls equals 0.82, PPYoloELoss/loss iou equals 0.23, PPYoloELoss/loss dfl equals 0.877, PPYoloELoss/loss equals 1.83, P@0.50 equals 8%, R@0.50 equals 98%, mAP@0.50 equals 0.68, and F1@0.50 equals 15% for all classes. It detects only nine classes, as shown in Fig. 18. Figure 19 shows a sample of predicted images from the YOLO-Nas model.

**Fig. 20:**
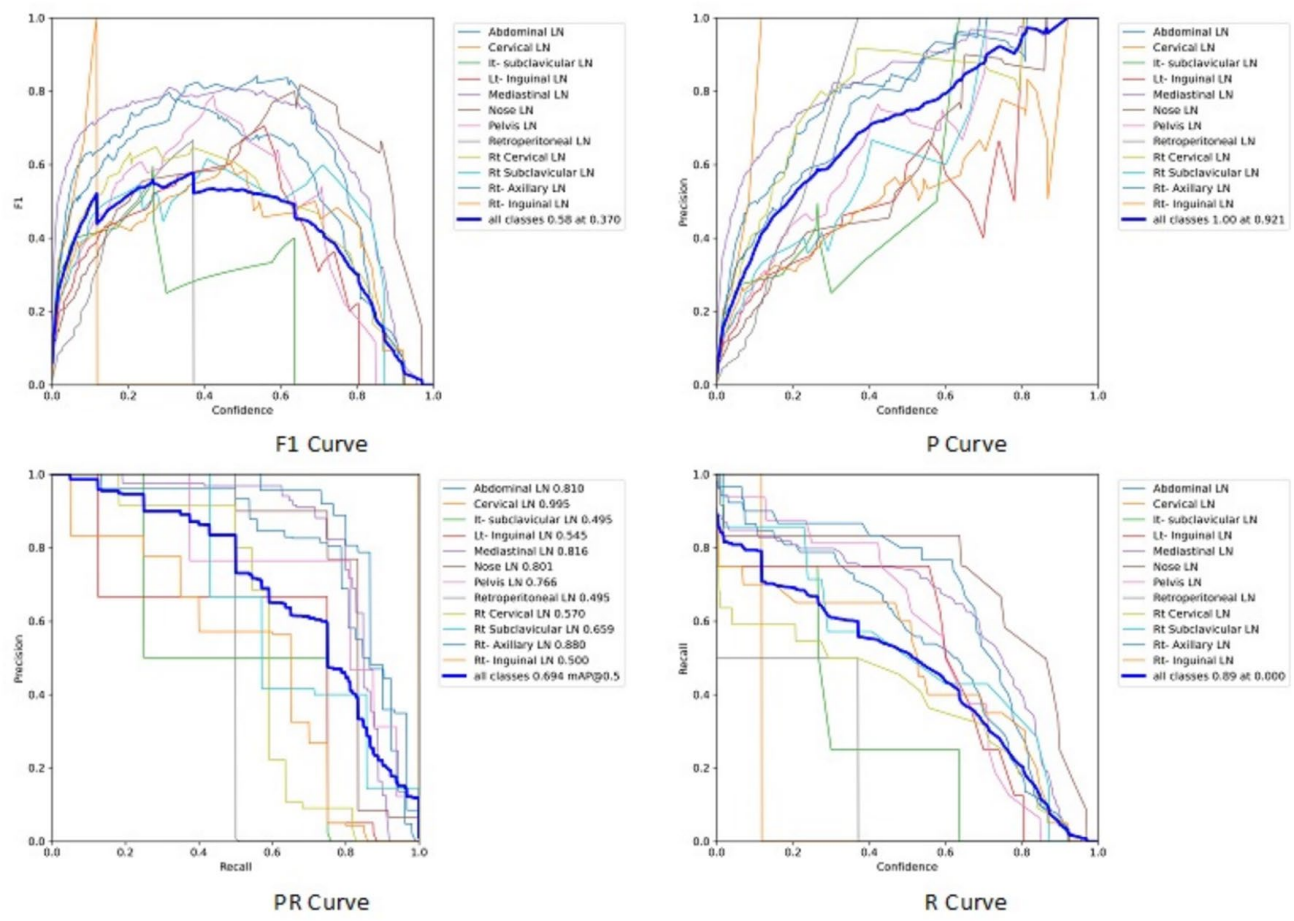
F1, P, PR, and R curves from the YOLOv7 model.

**Fig. 21:**
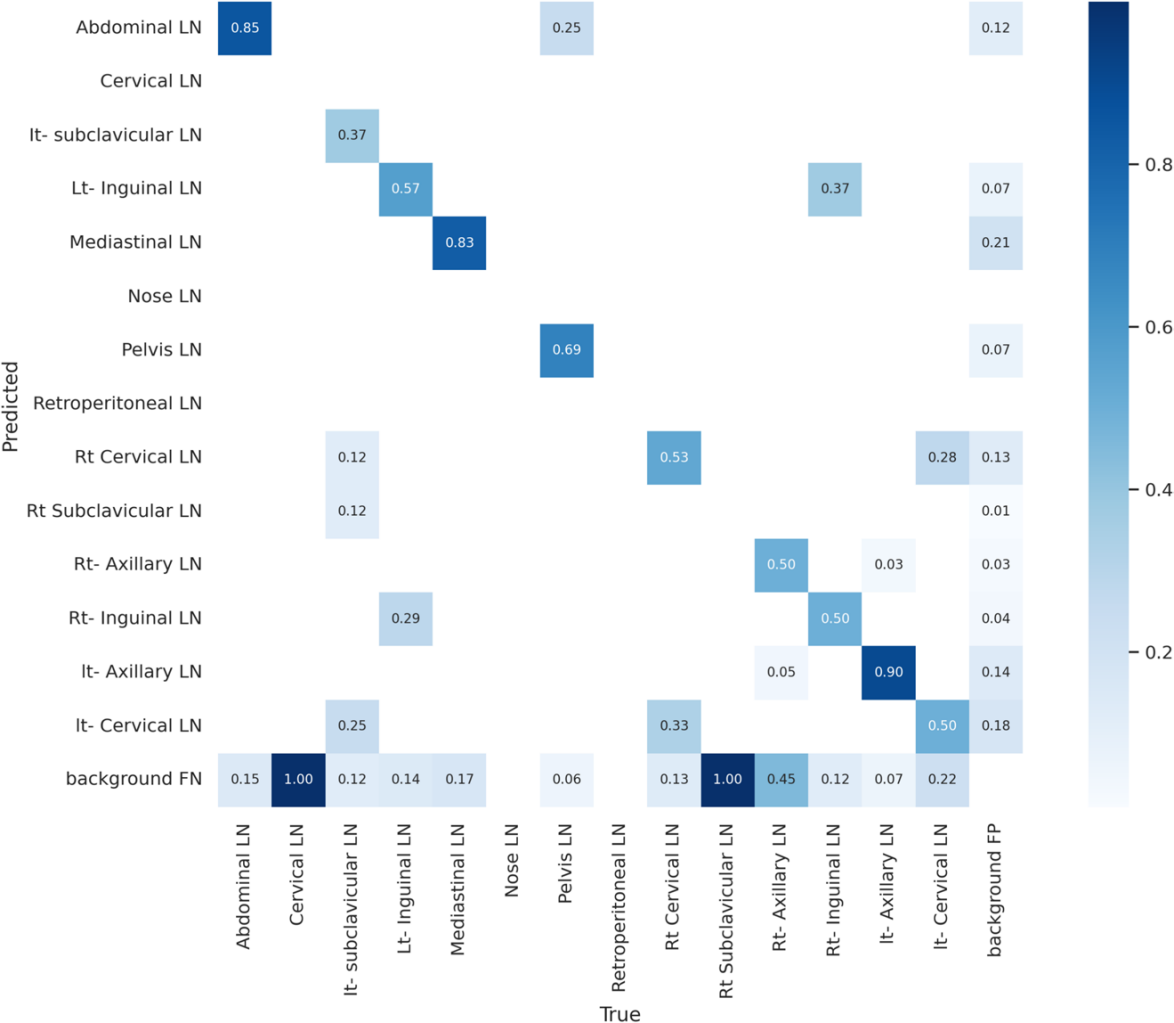
The confusion matrix of the YOLOv7 model.

**Fig. 22:**
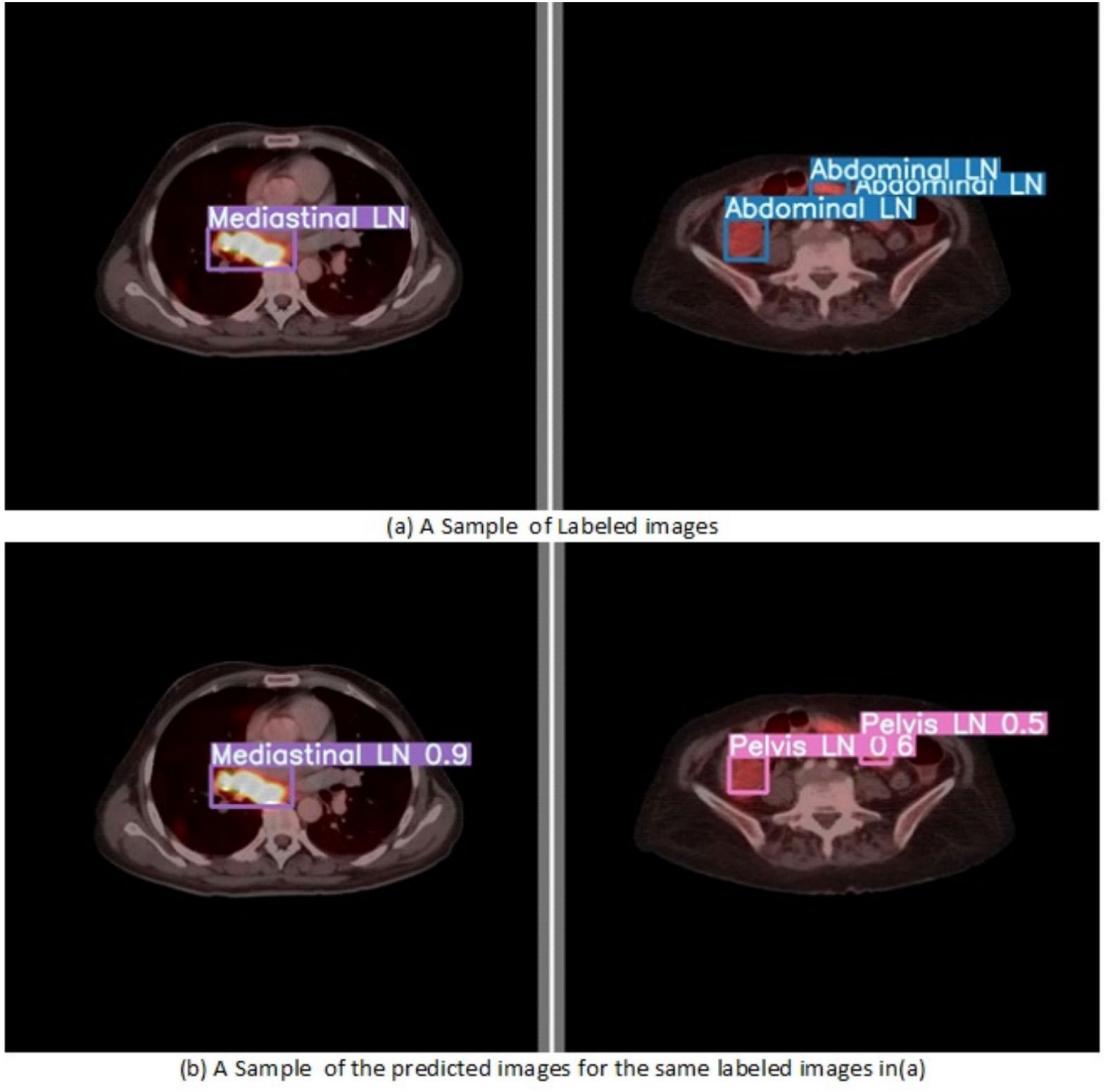
A sample of batch 0 containing (**a**) the labeled or annotated images for two images and (**b**) the predicted same two images as output from the YOLOv7 model for all classes of the LYE23 dataset.

For YOLOv7, we used the same configuration as YOLOv8, with 200 epochs. Figure 20 shows the resulting F1, P, PR, and R curves from the YOLOv7 model. Figure 21 shows the resulting CM of the YOLOv7 model. It detected only ten classes. Table 13 shows the P, R, and mAP@.5 results for all classes and the total. Figure 22 shows a sample of two annotated images from batch 0 and the same predicted images from the YOLOv7 model. Figure 7 shows the comparison among the four models, YOLO-Nas, YOLOv7, YOLOv8, and the modified YOLOv8 due to P, R, and mAP@0.5 measures that are recorded in the tables above.

**Table 13:** The YOLOv7 results of precision, recall, mAP50, and DSC for all classes of the LYN23 dataset.

Classes	Precision	Recall	mAP50	DSC
Abdominal	0.82	0.71	0.81	0.84
Cervical	1	0	1	1
It- subclavicular	0.32	0.25	0.50	0.60
Lt- Inguinal	0.47	0.75	0.55	0.70
Mediastinal	0.82	0.76	0.82	0.82
Pelvis	0.44	0.83	0.80	0.80
Nose	0.45	0.83	0.81	0.82
Rt Cervical	0.63	0.81	0.77	0.65
Rt Subclavicular	1	0.50	0.50	0.70
Rt- Axillary	0.92	0.50	0.57	0.80
Rt- Inguinal	0.54	0.57	0.66	0.62
lt- Cervical	0.47	0.65	0.50	0.55
Total	0.69	0.60	0.69	0.58

From Table 13, we observe that the YOLOv7 model detected the nose class but produced fewer results than the modified YOLOv8.

For YOLOv11, Table 14 shows the precision, recall, and mAP50 for all classes, along with the average values across all classes with 200 epochs. From Table 14, we can observe that the Normal class is the best detected class, as the precision, recall, and mAP50 are equal to 94%, 87%, and 84%, respectively. On the other hand, the worst detected class is the It-subclavicular class. It equals 47%, 50%, and 75% for precision, recall, and mAP50, respectively. Figure 24 shows the confusion matrix.

**Table 14:** YOLOv11 results of precision, recall, mAP50, and DSC for all classes of the LYN23 dataset.

Classes	Precision	Recall	mAP50	DSC
Abdominal	0.84	0.82	0.84	0.82
Cervical	0.49	1	1	0.65
It- subclavicular	0.47	0.50	0.75	0.48
Lt- Inguinal	0.78	0.75	0.77	0.76
Mediastinal	0.85	0.67	0.78	0.74
Normal	0.94	0.87	0.94	0.90
Pelvic	0.48	0.75	0.77	0.58
Rt Cervical	0.76	0.81	0.88	0.78
Rt Subclavicular	0.76	1	1	0.86
Rt- Axillary	0.91	0.62	0.85	0.73
Rt- Inguinal	0.79	0.71	0.83	0.74
lt- Axillary	0.64	0.91	0.92	0.75
lt- Cervical	0.89	0.54	0.71	0.67
Total	0.78	0.80	0.82	0.78

**Fig. 23:**
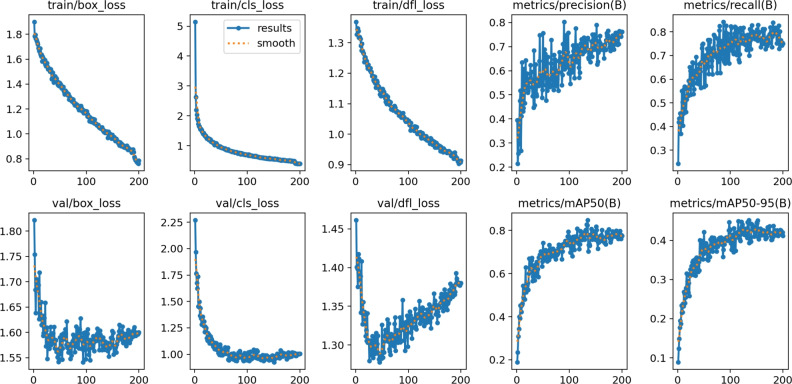
The training and validating loss matrices on the private dataset using YOLOv11.

Figure 23 shows the various loss matrices (box, cls, and dfl loss) in training and validation.

**Fig. 24:**
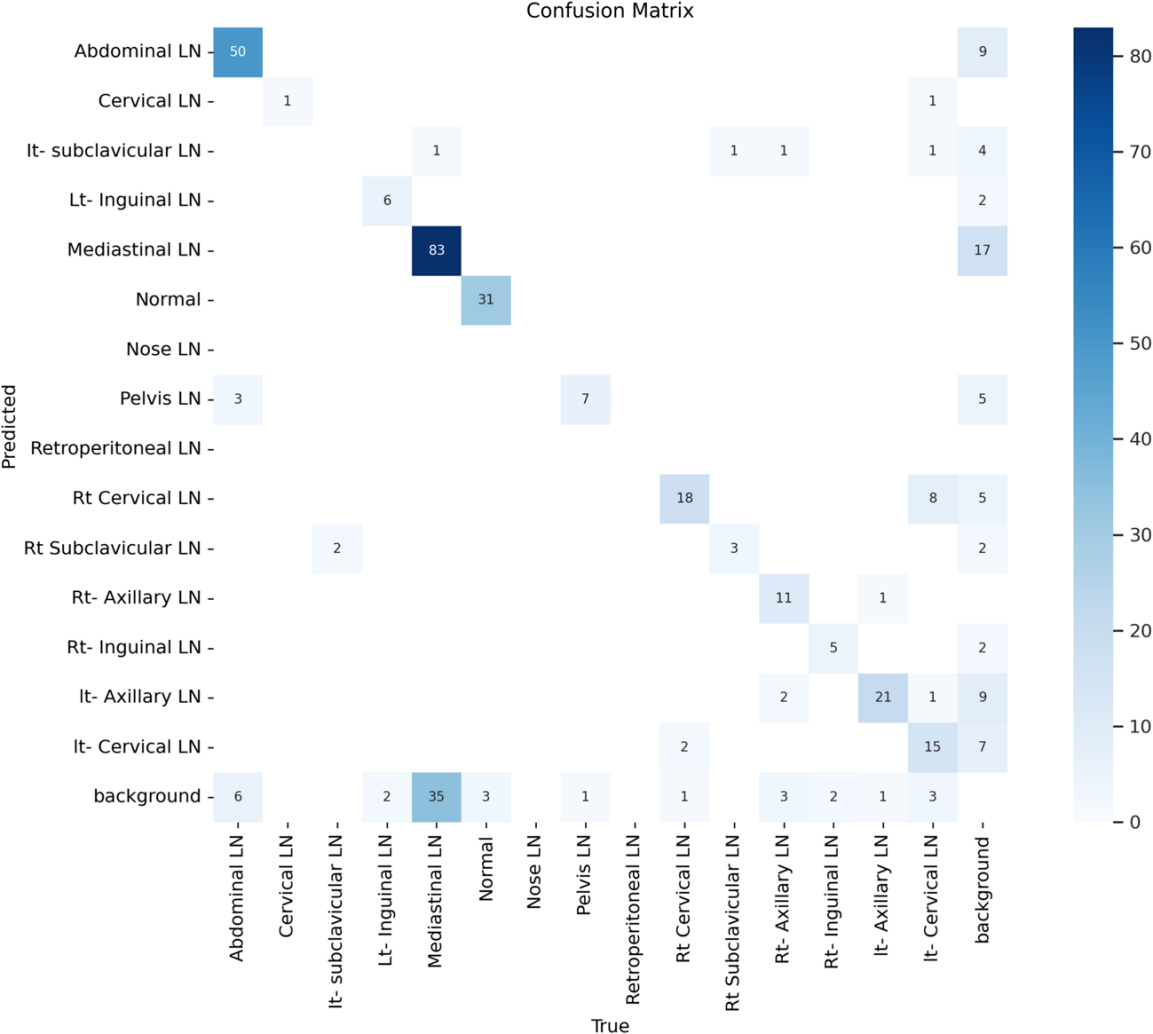
The confusion matrix on YOLOv11.

**Fig. 25:**
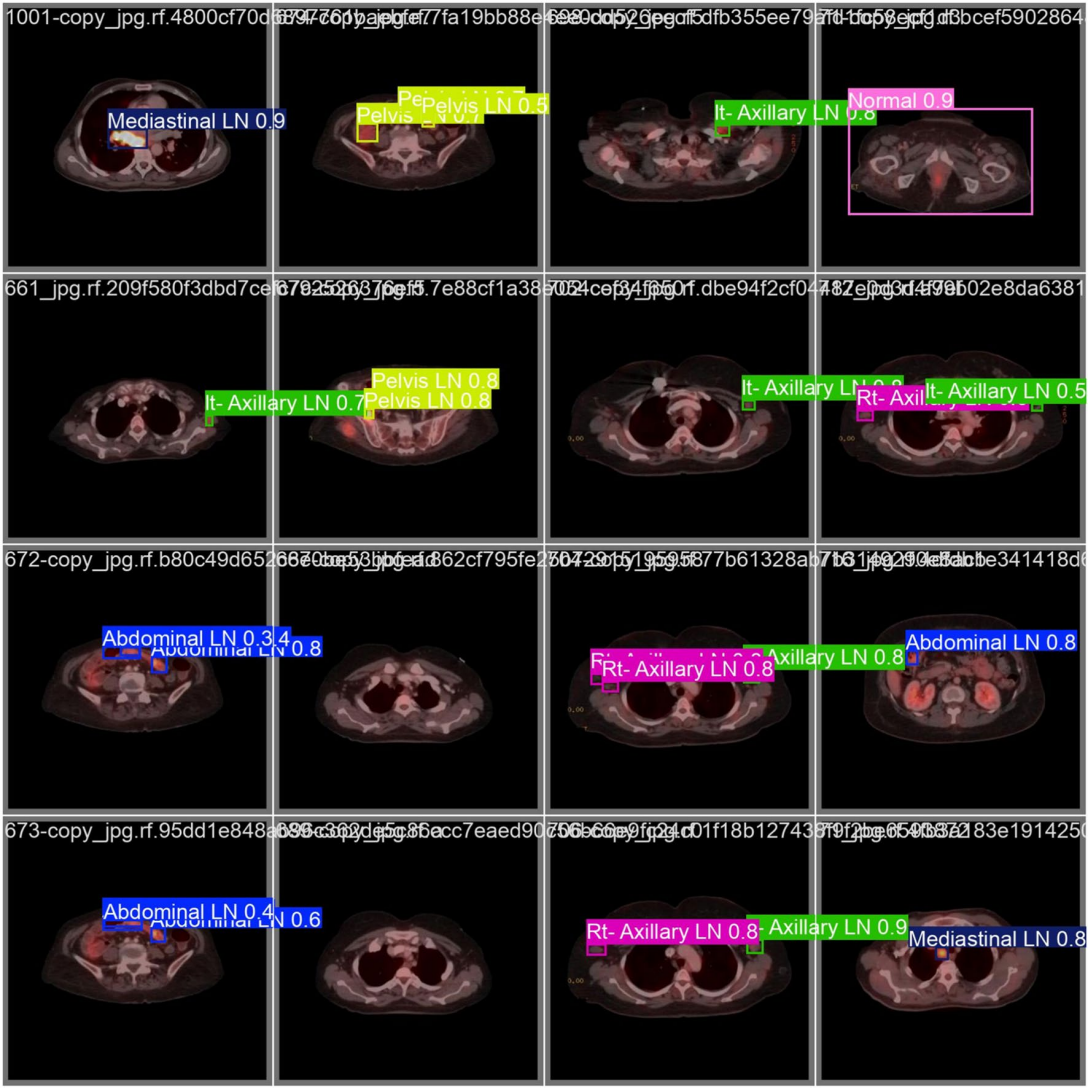
The batch sample of using YOLOv11 on the private dataset.

Figure 25 shows a sample batch prediction from the lymph node dataset using YOLOv11.

**Fig. 26:**
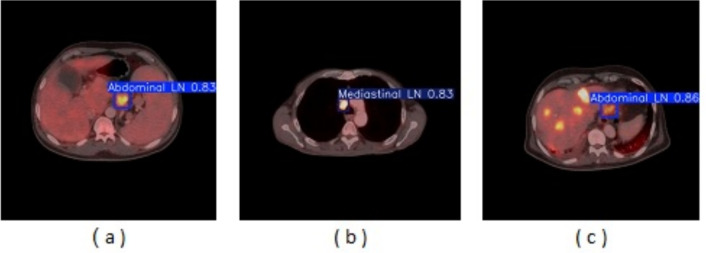
The LN predicted samples as an output of using YOLOv11 on the private dataset.

YOLOv11 does not detect some lymph nodes of the same characteristics in the same image as shown in Fig. 26c.

**Fig. 27:**
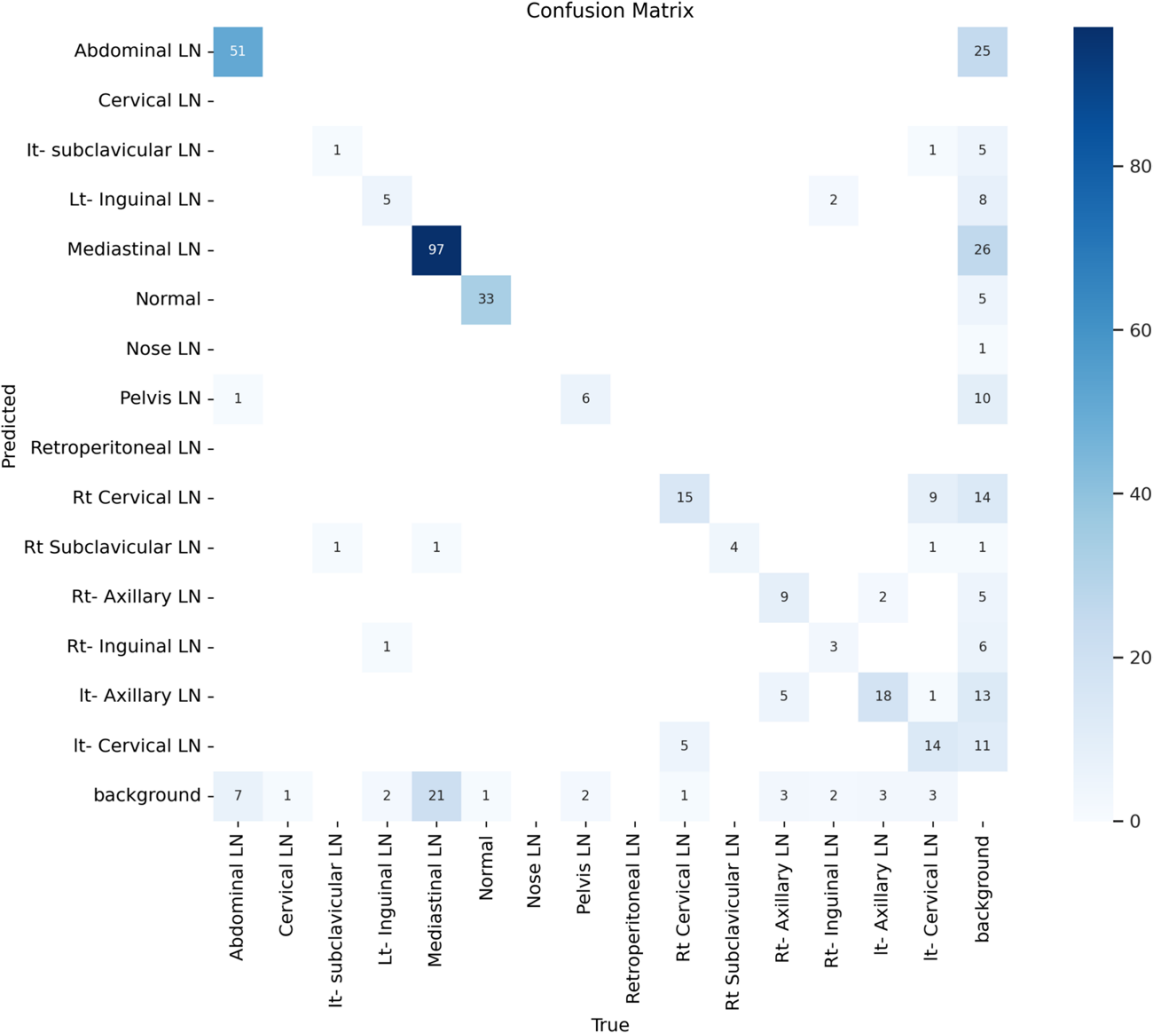
The confusion matrix on YOLOv12.

Figure 27 shows the confusion matrix of YOLOv12.

**Fig. 28:**
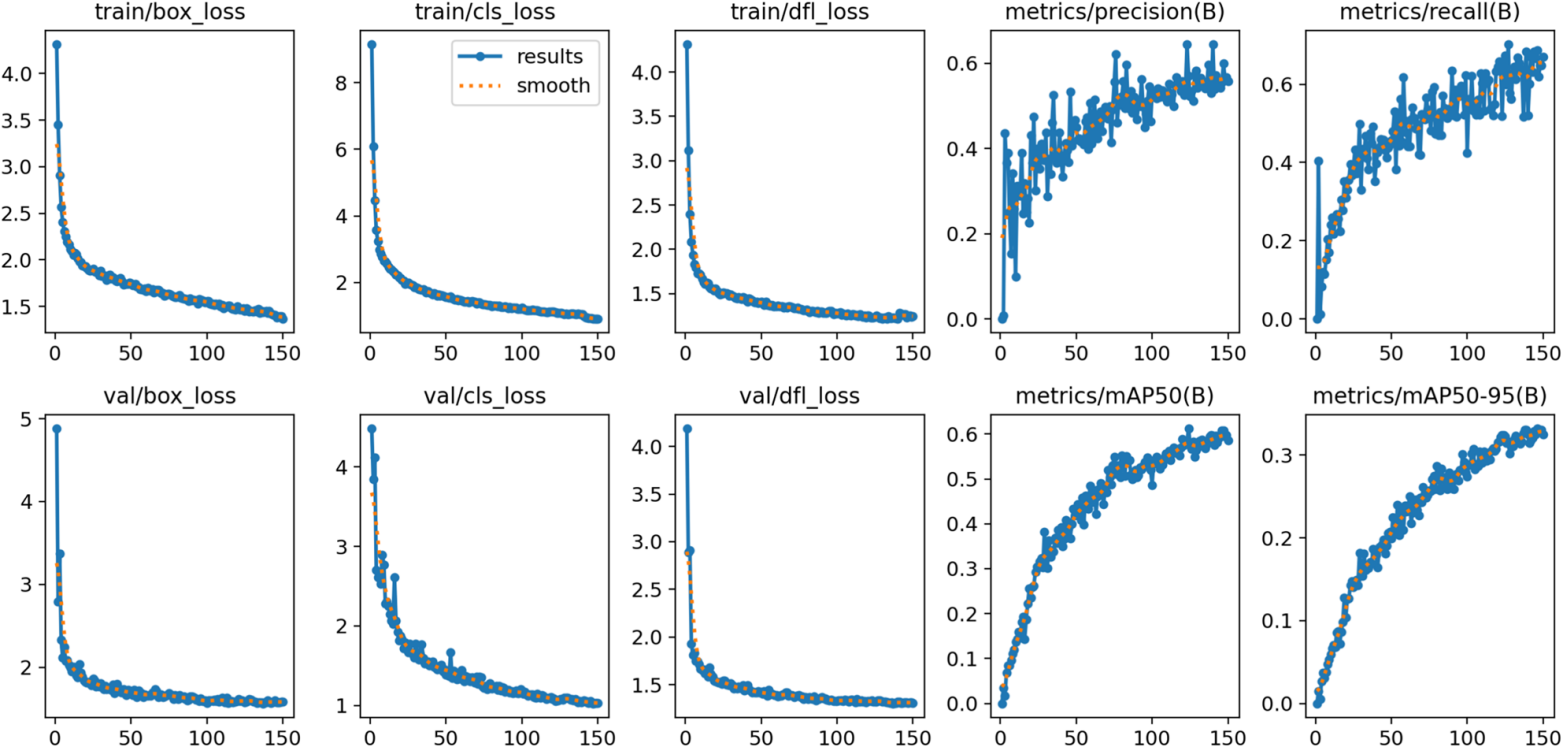
Training and validating loss matrices on the private dataset using YOLOv12.

For YOLOv12, Fig. 28 shows the various loss matrices (box, cls, and dfl loss) in the training and validation of YOLOv12. We stopped at epoch 150 as there are no changes in the other 50 epochs.

From Table 15 below, we present all results for the applied dataset. The modified YOLOv8 yields the best results among the other versions.

**Table 15:** Various approaches yield results of precision, recall, mAP50, and DSC for the private LYN23 dataset.

Method	Precision	Recall	mAP50	DSC
YOLOv7	0.69	0.60	0.69	0.58
YOLOv8	0.65	0.66	0.74	0.74
YOLOv9	0.68	0.79	0.77	0.73
YOLOv10	0.82	0.63	0.79	0.71
YOLOv11	0.78	0.80	0.82	0.78
YOLOv12	0.56	0.69	0.61	0.62
**The Modified YOLOv8 80/10/10**	0.75	0.83	0.85	0.77

From the previous table, we can see that YOLOv10 exceeds the presented modified YOLOv8 in precision by 7%, while the modified model exceeds it in recall and mAP50 by 20% and 6%, respectively. On the other hand, YOLOv11 is approximately equal to the results of the modified YOLOv8, but with less than 3% for both R and mAP50 and higher by the same 3% for P and DSC. In addition, YOLOv7, 8, and 12 give the worst results in our dataset. YOLOv9 yields results similar to theirs, but with less difference.

### Discussion

We predicted 13 classes where other studies predicted only one class, such as cervical LN in^22,23,26,29,30,56–60^, and^61^. Others predicted only pelvic LN, such as in^42^ and^43^. Other studies predicted only mediastinal LN, such as^34,46,62–64^, and^36^. We gathered about 1,931 images over the year and performed some data augmentation. Data augmentation was applied exclusively to the training set, while validation and test sets remained unmodified to prevent data leakage and ensure unbiased evaluation. and avoid falling in overfitting while others utilized only 117 to 200 images for training, such as^29^, and^59^ or 412 images, such as^56^, or between 30 and 71 such as in^31,57^, and^24^. Others utilized a small dataset from Table 1. Table 16 shows the comparison between the proposed modified Yolov8 model and other conducted studies that predicted lymph nodes classes using YOLO.

**Table 16:** A comparison among the proposed modified Yolov8 model and various conducted studies’ results.

Study	No. of Predicted classes	Utilized model	results	Dataset	Proposed model
Wang et al.^65^	cervical, lymphoma, and metastatic (3 classes)	Yolov7	mAP50=96.4%	up to 4580 sample	mAP50=100 for cervical class
Gnong et al.^66^	Pelvic lymph node (10 class)	YOLOv11	P is 88.5% and R is 71.3%	1,258 pelvic enhanced CT	P is 54%, R is 92%, and mAP50 is 87% for pelvic class
Li et al.^67^	Colon cancer (3 classes)	YOLOv8 and YOLOv12	P in YOLOv8 and 12 are 0.962 and 0.945; R for YOLOv8 and 12 are 0.896 and 0.907; and mAP@0.5 for YOLOv8 and 12 are 0.635 and 0.647.	1,847 images	detected about 13 classes of different datasets

YOLOv7 gives better accuracy in Wang et al.^65^, while the proposed model detected 13 classes and achieved 100% of mAP50 in the cervical class. In Gong et al.^66^, YOLOv11 achieves high accuracy while maintaining moderate computational efficiency. Its improved multi-scale feature extraction enables strong performance on small object detection tasks. Nevertheless, the model’s effectiveness is influenced by dataset-specific factors such as data distribution, object scale variability, and annotation quality. YOLOv8 provides a strong baseline with good detection capability, leveraging a PAN-FPN architecture for effective feature fusion. It achieves very fast inference speed while maintaining a balanced trade-off between accuracy and computational efficiency, as in Li et al.^67^.

The proposed approach focuses on target-specific lymph node detection, achieving strong performance by integrating a P6 layer into an extended PAN-FPN architecture. While this increases computational cost and reduces inference speed, it significantly improves detection across both small and large lymph nodes.

**Fig. 29:**
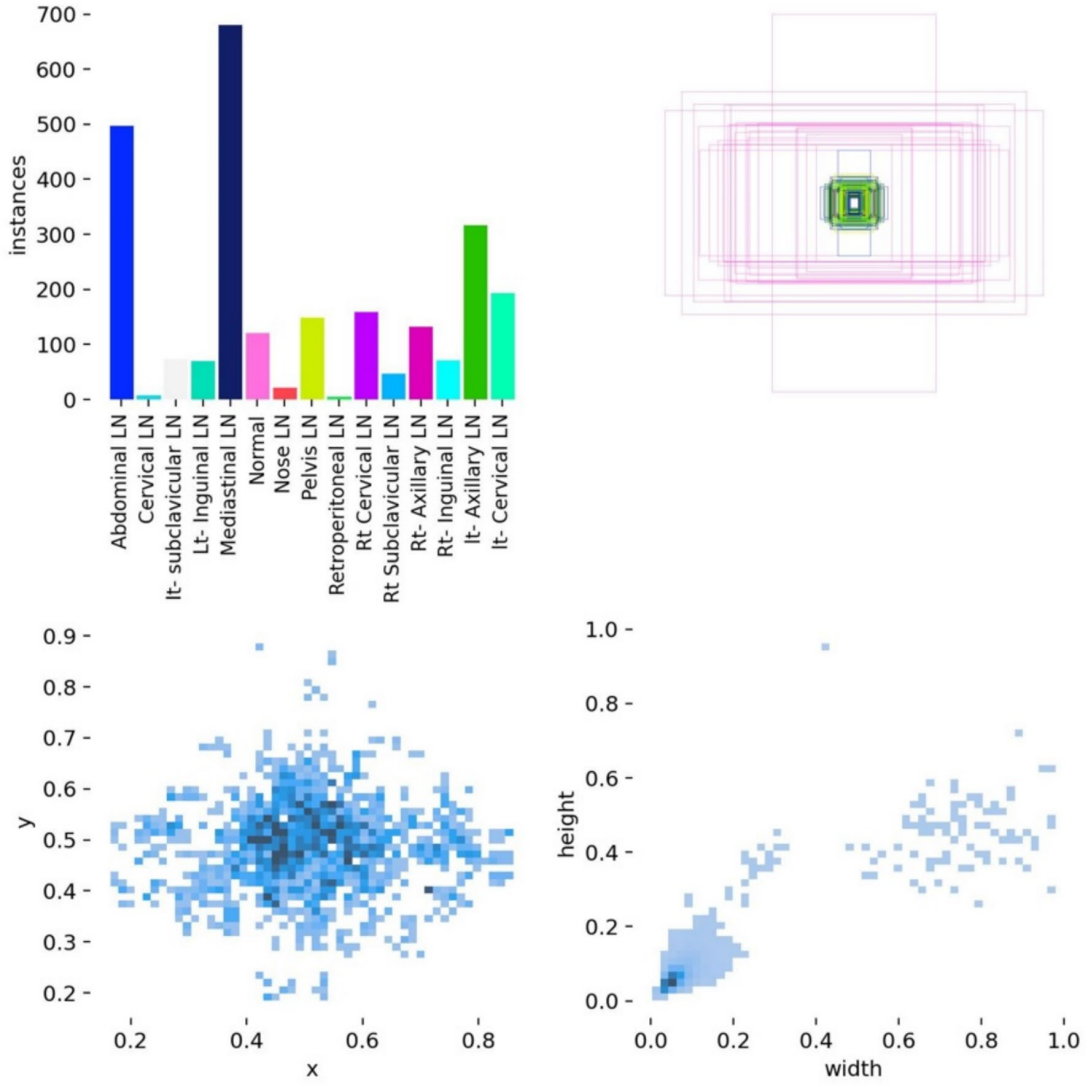
Top left: the number of instances for each class, Top right: Visualization of bounding boxes for target objects; Bottom Left: distribution of target center point coordinates (x, y); Bottom right: distribution of bounding box width and height.

We annotated 15 classes of 1931 images. Although YOLOv8 is a state-of-the-art model, it cannot predict the nose, retroperitoneal, and cervical classes in all 5 folds. Besides, YOLOv12 yields the worst results on our dataset, and none of the YOLO versions can detect the ”nose” and ”retroperitoneal” classes. That is because the number of instances is very low. The images that include the class ”nose” and ”retroperitoneal in the abdominal region” are about 14 and 9 images, respectively, in the whole dataset, as shown in Fig. 29.

The modified model employs larger convolutional kernels (7$\times$7), an extended detection scale (P6) for large-object detection, and increased channel depth up to 2048 to enhance the representation of deeper semantic features. These design choices improve the model’s ability to detect large and complex objects in the dataset. However, this improvement comes at the cost of increased computational complexity, resulting in higher FLOPs and memory consumption.

On the other hand, YOLOv12 added a background class. We balanced the number of images per class. When we resized the images to $400\times 400$, the model stopped at epoch No. 135 and did not complete 200 epochs. YOLOv8 augments the training data with Mosaic. Four different images are stitched together and fed into the model as input. This makes the model learn the actual objects from different positions and in partial occlusion. Therefore, we did not make mosaic augmentation in our dataset. We should use mosaic augmentation because we intend to make a general dataset for other models. YOLOv9, 10, 11, and 12 took less time than their predecessors.

We tried varying the number of epochs and the batch size in the adapted YOLOv8 and found that results improved when increasing the number of epochs to 200, but increasing the batch size beyond 16 did not affect the results in the last 50 epochs of training. The external validation remains limited.

Although the dataset was collected from five different centers within the same healthcare group, all sites follow a unified imaging protocol and scanner configuration. Therefore, the dataset reflects a unified study design in terms of protocol consistency. This may be challenging to generalize across other centers with different imaging systems, protocols, or patient populations, yet not impossible to implement.

Despite the promising performance of the proposed model in terms of quantitative metrics, it is important to emphasize that this study represents a methodological proof-of-concept rather than a clinically validated system. The model was evaluated under controlled experimental conditions using retrospective PET/CT data, and no prospective clinical evaluation or clinician-in-the-loop assessment was performed. Therefore, the current results do not directly reflect the model’s impact on clinical workflow, radiologist decision-making, or patient outcomes. In addition, practical deployment aspects such as integration with hospital PACS systems, inference time in real clinical settings, and robustness to imaging artifacts were not investigated in this study. Future work will focus on prospective clinical validation in collaboration with radiologists to assess the real-world applicability and clinical utility of the proposed system.

## Statistical analysis

We determined the baseline recall to be 0.80, the precision to be 0.78, and the mAP50 to be 0.82. For recall, mean ± std is 0.7043 ± 0.0873, and 95% confidence interval (CI) is (0.6235, 0.785). For precision, mean ± std is 0.7143 ± 0.0914, and 95% CI is (0.6297, 0.7988). For MAP50, mean v std is 0.7529 ± 0.0818, and 95% CI is (0.6772, 0.8285). In a one-sample t-test vs baseline, the t-statistic is -2.8994, and the p-value is 0.0274, indicating a statistically significant improvement. The precision t-statistic is -1.9013, and the p-value is 0.1060, indicating non-significance. The MAP50 t-statistic is -2.1718, and the p-value is 0.0729, which is not statistically significant.

In effect size (Cohen’s d vs baseline), recall Cohen’s d equals -1.0959, precision Cohen’s d equals -0.7186, and MAP50 Cohen’s d is -0.8209. They have a large effect. In the normality test (Shapiro-Wilk), p-values for recall, precision, and mAP50 are 0.9335, 0.3673, and 0.7920, respectively, indicating that the data appear to be normally distributed and that the t-test is valid. Based on the previous analysis, we conclude that the proposed modified YOLOv8 model demonstrates improved detection performance, with a mean mAP50 of 0.7529 ± 0.0818. Statistical analysis using a one-sample t-test indicates a significant improvement in recall (p = 0.0274), while precision and mAP50 show positive but non-significant trends. Despite this, large effect sizes across all metrics (Cohen’s d > 0.7) confirm strong practical improvements. Normality tests confirm the validity of parametric testing.

Comparing models with each other due to precision, recall, and mAP50.

- Precision: the best model is YOLOv10, then YOLOv11, and the modified YOLOv8 comes in the third order. The precision mean is 0.7043; the 95% CI is (0.6429, 0.7629).
- Recall: the modified YOLOv8 is the best model, followed by YOLOv11, and YOLOv9 comes in third order. The mean recall is 0.7143, and the 95% CI is (0.6529, 0.7771).
- mAP50: the modified YOLOv8 is the best model, then YOLOv11, and in the third order is YOLOv10. The mAP50 mean is 0.7529, and the 95% CI is (0.6929, 0.8043).

## Conclusion

Assessing LN status is crucial for determining appropriate treatment strategies. Radiologists primarily rely on image-based evaluations for preoperative LN assessment, which can be time-consuming and error-prone. Inaccurate LN status evaluation may lead to either undertreatment or overtreatment, raising the risk of recurrence and postoperative complications. On the contrary, we propose an object detection method to localize the 13 LNs located in different body parts; meanwhile, many imaging techniques fail to assess the small LNs. To generate and acquire fusion PET-CT images, we first established a dataset based on actual patient cases, then collected their CT and PET images for fusion. The noisy PET-CT images were cleaned and annotated, yielding 15 labels/classes. After that, Data augmentation was applied exclusively to the training set, while validation and test sets remained unmodified to prevent data leakage and ensure unbiased evaluation. We completed this module by dividing the dataset into three sets: training, validation, and testing. Secondly, we modified YOLOv8 by replacing the backbone with a different kernel, adding one CONV block, and optimizing various hyperparameters. We compared eight one-stage techniques using various performance measures: YOLOv7, YOLOv8, YOLOv9, YOLOv10, YOLOv11, YOLOv12, YOLO-Nas, and the modified YOLOv8. 15 classes were annotated in the dataset, but only 13 classes were finally detected by the proposed model. In the future, we will accurately annotate and collect additional images to clarify the nose class and expand the dataset. On the other hand, we will modify and combine YOLOv11 with other object detectors.

## Data Availability

This research study was conducted retrospectively using subject data made available in open access by [organization or entity: https://universe.roboflow.com/mansoura/lymph-nodes/browse].

## abbreviations

AI: Artificial Intelligence
PET-CT: A Positron Emission Tomography - Computed Tomography scan
MAP: Mean Average Precision
CNNs: Convolutional Neural Networks
FDG: Fluorodeoxyglucose
SUVs: Standard Uptake Values
LNM: Lymph Node Metastasis
LT , RT: Left , Right
GBM: Gradient Boosting Machine
PPV: Positive Predictive Value
CTV: Clinical Target Volumes
ROI: The region of interest
LRM: Logistic Regression Model
FCN: Fully Convolutional Network
DT: Decision Tree
ANN: Artificial Neural Network
RFE: Recursive Feature Elimination
FPN: Feature Pyramid Network
DSC: Dice Similarity Coefficient
PPV: Positive Predictive Value
PR: Precision Recall
ALN: Axillary Lymph Nodes
LASSO: Least Absolute Shrinkage and Selection Operator
LVI: Lymph vascular Invasion
HL: Hepatic Lymphoma
YOLOv8: You Only Once version 8
CONV: Convolutional
DCN: Dynamic Circuit Network
cos-lr: Cosine Learning Rate
SPP: Spatial Pyramid Pooling
Bbox: Bounding box
ALL: Acute Lymphoblastic Leukemia
LN: Lymph Node
DSC: Dice Similarity Coefficient
DL, ML: Deep Learning , Machine Learning
US , MRI: Ultrasound, Magnetic Resonance Imaging
FNAC: Fine Needle Aspiration Cytology
AUC: Area Under the Curve
EMR: Electronic Medical Records
HNSCC: Head and Neck Squamous Cell Carcinoma
RF: Random Forests
NPV: Negative Predictive Value
SVM: Support Vector Machine
DLN: Deep Learning Nomogram
DNN: Deep Neural Network
NLP: Natural Language Processing
LR: Logistic Regression
ICCA: Intrahepatic cholangiocarcinoma
PLND: Pelvic Lymph Node Dissection
LYE23: Lymph node data set
MAP: Mean Average Precision
IOU: Intersection Over Union
BMC: Baseboard Management Controller
ALK: Anaplastic Lymphoma Kinase
CRC: Colorectal Cancer
CECT: Contrast-Enhanced Computer Tomography
CEA: Carcino Embryonic Antigen
DICOM: Digital Imaging and Communications in Medicine
FPN: Feature Pyramid Network
Lr0 , Lrf: Initial, Final Learning Rate
SPPF: Spatial Pyramid Pooling Fusion
FLOPs: floating-point operations
CLS: Classification Loss
IOU: Intersection over Union
